# Predictors of anaemia in mothers and children in Uttar Pradesh, India

**DOI:** 10.1017/S1368980024000028

**Published:** 2024-01-08

**Authors:** Leila M Larson, Tinku Thomas, Anura V Kurpad, Reynaldo Martorell, John Hoddinott, Victoria Oluwapamilerin Adebiyi, Sumathi Swaminathan, Lynnette M Neufeld

**Affiliations:** 1 Department of Health Promotion, Education, and Behavior, Arnold School of Public Health, University of South Carolina, 915 Greene Street, Columbia, SC 29208, USA; 2 Department of Biostatistics, St John’s Medical College, Bangalore, India; 3 Department of Physiology, St John’s Medical College, Bangalore, India; 4 The Hubert Department of Global Health, Rollins School of Public Health, Emory University, Atlanta, USA; 5 Division of Nutritional Sciences, Cornell University, Ithaca, NY, USA; 6 Division of Nutrition, St John’s Research Institute, Bangalore, India; 7 Food and Agriculture Organization of the United Nations (FAO), Rome, Italy

**Keywords:** Anaemia, infection, inflammation, hemoglobin, iron

## Abstract

**Objective:**

Anaemia affects more than half of Indian women and children, but the contribution of its causes remains unquantified. We examined interrelationships between Hb and nutritional, environmental, infectious and genetic determinants of anaemia in non-pregnant mothers and children in Uttar Pradesh (UP).

**Design:**

We conducted a cross-sectional survey of households in twenty-five districts of UP between October and December 2016. We collected socio-demographic data, anthropometry and venous blood in 1238 non-pregnant mothers and their children. We analysed venous blood samples for malaria, Hb, ferritin, retinol, folate, Zn, vitamin B_12_, C-reactive protein, *α*1-acid glycoprotein (AGP) and *β*-thalassaemia. We used path analysis to examine pathways through which predictors of anaemia were associated with Hb concentration.

**Setting:**

Rural and urban households in twenty-five districts of UP.

**Participants:**

Mothers 18–49 years and children 6–59 months in UP.

**Results:**

A total of 36·4 % of mothers and 56·0 % of children were anaemic, and 26·7 % of women and 44·6 % of children had Fe deficiency anaemia. Ferritin was the strongest predictor of Hb (*β* (95 % CI) = 1·03 (0·80, 1·27) g/dL in women and 0·90 (0·68, 1·12) g/dL in children). In children only, red blood cell folate and AGP were negatively associated with Hb and retinol was positively associated with Hb.

**Conclusions:**

Over 70 % of mothers and children with anaemia had Fe deficiency, needing urgent attention. However, several simultaneous predictors of Hb exist, including nutrient deficiencies and inflammation. The potential of Fe interventions to address anaemia may be constrained unless coexisting determinants are jointly addressed.

Anaemia affects 40 % of children 6–59 months of age and 30 % of non-pregnant women of reproductive age globally^(1)^. The burden is particularly high in low- and middle-income countries and has important implications for cognition, school achievement, work productivity and income generation. The World Health Assembly set a target for 2025 of reducing anaemia by 50 % relative to the 2010 prevalence in women worldwide. To achieve this target, interventions must address the causes of anaemia appropriately. It is commonly stated that 50 % of anaemia is caused by Fe deficiency anaemia^(2)^; however, the aetiology of anaemia is multifactorial and varies by setting, and the proportion of anaemia that is amenable to Fe treatment is still not well understood^(3,4)^.

The determinants of anaemia have been well characterised in the literature and include a complex pattern of biological, socio-demographic, environmental and genetic factors^(5)^. Assessments of anaemia in vulnerable populations, and interventions and policies to address anaemia, have attempted to map its prevalence and understand its causes. The most important anaemias result from nutritional deficiencies and parasitic infections that cause increased loss or reduced absorption or utilisation of Fe (soil-transmitted helminths, malaria, schistosomiasis and other parasitic infections), and genetic disorders affecting Fe or Hb metabolism^(5)^. Many studies have examined associations between anaemia or Hb concentration and their predictors using bivariate analyses^(3,6–10)^. However, the interplay of direct and indirect associations between Hb and its multiple predictors is inadequately understood. This information is critical for the design of programmes that can effectively reduce the burden of anaemia. The current analysis seeks to further elucidate the aetiology of anaemia, by examining the interactions and associations between genetic, environmental, infectious and nutritional predictors of Hb concentration in women and children living in rural and urban areas of Uttar Pradesh state, India. Uttar Pradesh is located in Northern India and shares a border with Nepal. It is one of the largest states in India with some of the highest numbers of malnourished women and children. According to the National Family Health and Welfare Survey-4, conducted prior to this study, 46·3 % of children under 5 years of age were stunted and 63·2 % were anaemic, and 52·5 % of non-pregnant women of reproductive age were anaemic^(11)^.

## Subjects and methods

### Study design and sampling strategy

This study was led by the Global Alliance for Improved Nutrition with partner organisations St John’s Research Institute, Bangalore, India (SJRI), The India Nutrition Initiative and Cornell University. The study consisted of a cross-sectional survey of non-pregnant mothers of reproductive age (MRA) 18–49 years of age and children 6–59 months of age (preschool-age children (PSC)) living in rural and urban Uttar Pradesh. A total of twenty-five districts were selected: ten districts were purposefully sampled as part of a baseline evaluation to a double fortified salt programme in the state^(12)^ (details forthcoming) and fifteen additional districts were selected randomly using simple random sampling with a random number generator out of all remaining districts in the state, for a total of twenty-five districts. Within each district, five villages (rural) and five wards (urban) were randomly selected from the 2011 Census of India. Within each ward, one Census Enumeration Block was selected randomly using the 2011 Census of India. Within each of these villages and Census Enumeration Blocks, five households were selected to be included in the survey using a modified random walk method (see online supplementary material, Supplemental Fig. 1).^(13)^ In nine wards, an insufficient number of households fit our inclusion criteria (i.e. there were fewer than five households containing a mother of reproductive age with at least one child 6–59 months of age). In these cases, where possible, we attempted to select additional households from neighbouring villages or wards selected for the survey in the same district. As such, the total number of participants included in the study varied slightly from the original design. Within each household, one non-pregnant mother of reproductive age and one of her PSC 6–59 months of age were included. Households were excluded only in the case of physical or mental impairment that could impede measurements or provision of informed consent. Data were collected between October and December 2016.

A sample size of 1250 households in total was calculated to obtain a 5 % precision on the prevalence of Fe deficiency, in twenty-five randomly selected districts, anticipated prevalence of 25 %^(14)^, average household size of 5·7^(11)^ and a design effect of 2^(15)^. Using this pre-specified sample size of 1250, for this analysis, we were able to detect a minimum correlation coefficient with Hb of 0·09, with 90 % power and 5 % precision.

### Measurements and data collection

All MRA were interviewed with a household survey. The survey consisted of questions about household demographic characteristics such as socio-economic status, water, sanitation, hygiene, use of Integrated Child Development Services (a programme delivered through the Anganwadi Centres), use of other safety net programmes, as well as information on the mother and child’s medical history and use of nutritional supplements (i.e. Fe, folic acid, Zn, Ca, vitamin C, vitamin D and multivitamins). Women’s dietary diversity was assessed based on a 9-food group score for a 24-h recall period^(16)^ and for children a 7-item child dietary diversity score was assessed according to WHO guidelines at the time during a 24-h recall period^(17)^. Food insecurity in the past 12 months was measured using an 8-item Food Insecurity Experience Scale^(18)^.

Plax-Cruzer Series (Indman Scale Corporation) weighing scales were used to assess weight of the mother and child to the nearest 0·1 kg, using the tare function for child weight. Standing height was taken from MRA and children 2 years and above using the Seca 213 stadiometer to the nearest 0·1 cm. Recumbent length of children less than 2 years of age was measured using the Seca 417 infantometer (Seca) to the nearest 0·1 cm. Stadiometers, length boards and weighing scales were calibrated daily using standard procedures provided by the manufacturers. Reliability estimates for anthropometric measurements were obtained by comparing the supervisors’ measurements (only supervisors performed anthropometry measurements) to those of an expert when assessing the same child, using five children^(19,20)^. Reliability measurements for weight and height yielded a Pearson’s correlation coefficient between measurements of the data collectors and expert of > 0·98 and a coefficient of reliability of > 0·98.

Hb concentration was measured using the HemoCue 201 + Analyser (HemoCue). A non-fasting venous blood sample (10 mL) was collected from each MRA and 7 mL was collected from each PSC. First, 4 mL of venous blood was collected in plastic spray-coated K_2_EDTA tubes from BD Vacutainer^®^. After slowly inverting the K_2_EDTA vial twice, a syringe was used to collect a few drops of whole blood from the tube and to place them on a clean plastic slide. The phlebotomist then filled a microcuvette with the drop and immediately tested the Hb concentration using the HemoCue. A drop of whole blood was also used to test for the presence of malaria, specifically P. vivax and P. falciparum, using a rapid diagnostic kit (FalciVax immune-chromatography, Zephyr Biomedicals) on both women and children.

An additional 3–6 mL of venous blood was collected in trace-element free tubes, with increased silica act clot activator, silicone-coated interior from BD Vacutainer®.

### Laboratory blood measurements

Laboratory blood analyses were conducted at SJRI (i.e. red blood cell folate) and Sanjay Gandhi Postgraduate Institute (SGPGI) (i.e. all other analyses). Internal laboratory quality control was performed according to standard procedures. The laboratory at SGPGI completed the RIQAS certification through Randox Laboratories Limited (United Kingdom). The laboratory at SJRI participated in the Centers for Disease Control and Prevention’s VITAL-EQA programme.

Serum ferritin and vitamin B_12_ were measured using sandwich electrochemiluminescence immunoassay ELECSYS 2010 (ROCHE Diagnostics). Serum C-reactive protein (CRP) and *α*1-acid glycoprotein (AGP) were measured using immunoturbidimetry: Cobas Integra 800 for CRP and Hitachi 902 for AGP (ROCHE Diagnostics). Serum Zn was measured using atomic absorption spectroscopy Hitachi Model Z2300 (Hitachi Group). *β*-thalassaemia was measured using ion-exchange HPLC VARIANT II *β*-thalassaemia Short Programme (BIO-RAD Laboratories). Serum retinol was measured using reverse-phase HPLC: Dionex Ultimate 3000 series HPLC instrument (Thermo Fisher Scientific). Red blood cell (RBC) folate was quantified using the WHO-recommended folate microbiological assay^(21)^. The folate concentration was determined by measuring the turbidity of the inoculated medium at 590 nm using a microplate reader (SYNERGY H1 microplate reader, BioTek Instruments). The assay was carried out in a 96-well microplate and calibrated using 5-methyltetrahydrofolic acid standard provided by the CDC^(22,23)^.

### Variable creation and statistical analyses

A Wealth Index, using five categories, was calculated using principal component analysis with family assets, house materials and number of rooms, and agricultural land ownership, home ownership or commercial ownership. A water, sanitation and hygiene (WASH) index, also using quintiles, was calculated using principal component analysis with improved or unimproved source of drinking water, toilet facility and hygienic appearance of the mother and her child and the household compound^(24)^. BMI for women was derived by dividing the weight of mothers (in kg) by the square of their height (in cm). The following BMI categories were used to classify women: < 18·5, 18·5–24·9, 25·0–29·9 and over > 30 kg/m^2^. Length-for-age, weight-for-length and weight-for-age z-scores were calculated using the WHO 2006 child growth standards. Z-scores less than –2 were used to define stunting, wasting and underweight, respectively, and z-scores less than –3 were used to define severe stunting, severe wasting and severe underweight^(19)^.

Anaemia was defined according to WHO criteria^(25)^ in MRA: any anaemia as Hb < 12 g/dL, mild anaemia as Hb 11–11·9 g/dL, moderate anaemia as Hb 8–10·9 g/dL and severe anaemia as Hb < 8 g/dL; in PSC, any anaemia as < 11 g/dL, mild anaemia as Hb 10–10·9 g/dL, moderate anaemia as Hb 7–9·9 g/dL and severe anaemia as Hb < 7 g/dL. Hb adjustment for altitude was not needed as all areas were below 1000 m above sea level^(25)^. Fe deficiency was defined as ferritin < 15 µg/L in MRA and < 12 µg/L in PSC^(26)^. Vitamin A deficiency was defined as serum retinol concentration < 0·7 µmol/L in MRA and PSC^(27)^. Ferritin (children and women) and retinol (children only) were adjusted for inflammation (CRP and AGP) using the BRINDA regression approach^(28,29)^. Vitamin B_12_ deficiency was defined as vitamin B_12_ concentration < 150 pmol/L in MRA and PSC^(30)^. Zn deficiency was defined as Zn concentration < 10·7 µmol/L in MRA and < 9·9 µmol/L in PSC^(31)^. Individual Zn values were adjusted to a non-fasting state and 20 min between collection and processing for both women and children^(32)^. In children only, they were also adjusted for CRP and AGP using the BRINDA regression approach^(32)^. RBC folate level to define folate deficiency (for all age groups) using macrocytic anaemia as haematological indicator was defined as < 305 nmol/L^(33)^. Elevated CRP was defined as CRP concentration > 5 mg/L^(34)^. Elevated AGP was defined as AGP concentration > 1 g/L^(35)^. *β*-thalassaemia was defined as Hb A2 variant > 3·2 %^(36)^.

We compared the magnitude of the associations between various predictors by fitting the data to a pre-specified model to determine their association with one another and with Hb (see online supplementary material, Supplemental Fig. 2). The pre-specified model was based on a review of the literature, and the pathways leading to anaemia were proposed drawing on the UNICEF conceptual framework of malnutrition that highlights the immediate, underlying and basic causes of malnutrition^(37)^. Explanations of the theory behind the model development are presented in online supplementary material, Supplemental Fig. 2.

Univariate and bivariate analyses were conducted using SAS 9.4 (SAS Institute), and the path model was developed in StataSE version 15 (StataCorp) using maximum likelihood estimation. First, means, standard deviations and prevalence statistics were derived using standard SAS procedures. Non-normally distributed blood biomarkers (ferritin, vitamin B_12_, RBC folate, Zn, retinol, CRP, AGP) were natural logarithm (ln) transformed and geometric means were reported. Second, multiple variable logistic regression models were used to estimate the factors associated with anaemia in children and women, and population attributable fractions were calculated for each of these factors^(38)^. Variables from our pre-specified theoretical model (see online supplementary material, Supplemental Fig. 2) were included in the multiple variable models. In all analyses, we accounted for clustering at the district level and weighted for district and setting (urban and rural) sampling. Weights were constructed for rural and urban populations separately using adjustment factors for (1) the proportion of people living in rural and urban localities in each district, (2) the size of each district and (3) the district’s likelihood of being selected. In SAS, proc surveymeans/surveyfreq and proc surveyreg/surveylogistic commands were used to account for the complex survey design; in StataSE, the svy command was used.

Third, the hypothesised path model examined the direct and indirect associations among WASH, dietary diversity, food insecurity, CRP, AGP, nutritional biomarkers (ferritin, Zn, retinol, vitamin B_12_, RBC folate) and Hb concentration. Prior to running the path model, Pearson’s correlations were run to examine correlations between all variables included in the model. A single model for PSC and for MRA was fitted to the data. Model fit was evaluated with common standards: a comparative fit index^(39)^ and a Tucker Lewis index > 0·90 for acceptable fit and > 0·95 for good fit^(40)^, and a standardised root mean squared residual^(41)^ and a root mean square error of approximation < 0·08 for acceptable fit and < 0·05 for good fit^(42)^. If model fit statistics were not acceptable, modification indices were examined to decide whether additional pathways should be examined to improve model fit, and the model was re-specified. The PSC model was adjusted for child age in months. Group comparison for rural and urban settings was used to determine whether the model may be different in the two populations. A Wald test was used to determine statistically significant (*P*-value < 0·05) differences for any particular path. Standardised and unstandardised estimates were calculated.

*β*-thalassaemia trait was measured in only a subset of the women (*n* 744) due to insufficient blood, and the prevalence of *β*-thalassaemia trait was low in this population (3 %); hence, a path analysis with *β*-thalassaemia was run separately. The model with thalassaemia included direct pathways from *β*-thalassaemia trait to Hb concentration and to ferritin concentration.

A further additional analysis was run using full information maximum likelihood. Several laboratory analyses were missing for some participants, particularly children, given insufficient blood sample. Therefore, 45 % of PSC and 19 % of MRA were missing at least one value for the predictors of interest. Additional path modelling was done in StataSE using the sem command and the mlmv option, which estimates coefficients using full information maximum likelihood with all available data under the assumption that any missing data were at random^(43)^.

## Results

Across the state, 34·5 % of mothers and 13·8 % of fathers had no schooling; mean household size was 6·7 in urban and 7·5 in rural settings (Table 1). In PSC, mean dietary diversity for the 24-h period was poor and fewer than 5 % of children consumed flesh foods (Table 2). Similarly, in mothers, dietary diversity was low and fewer than 8 % of women consumed meat or fish (Table 3). Prevalence of stunting, wasting and underweight in PSC was 47·7, 6·7, and 32·5 %, respectively, and 25·9 % of MRA had a BMI < 18·5 kg/m^2^ (Tables 2 and 3). Prevalence of anaemia was 56·0 % in PSC and 36·4 % in MRA; prevalence of low ferritin, or Fe deficiency, was 64·3 % in PSC and 51·0 % in MRA after adjusting for inflammation. Among those with anaemia, 78·1 % of PSC and 74·4 % of MRA had Fe deficiency. Among those with Fe deficiency, 68·0 % of PSC and 50·6 % of MRA had anaemia. Malaria was < 1 %, likely due to the survey being conducted in the non-malarial months (October–December) (Tables 2 and 3); therefore, malaria was not included in further analyses.

**Table 1:** Descriptive characteristics of non-pregnant mothers of reproductive age and preschool-age children living in Uttar Pradesh^*^

		State survey sample (*n* 1238)			Urban state survey sample (*n* 582)			Rural state survey sample (*n* 656)		
		Mean or %	*n*	sd	Mean or %	*n*	sd	Mean or %	*n*	sd
Child characteristics	Age									
	6–23 months	30·9	395		31·6	195		30·8	200	
	24–59 months	69·1	826		68·4	384		69·2	442	
	Age in months	32·0		14·1	30·6		8·9	32·3		18·0
	Gender									
	Girls	40·6	534		44·5	263		39·8	271	
Family characteristics	Religion									
	Hindu	86·4	1022		70·0	428		90·1	594	
	Muslim	13·0	207		27·9	146		9·7	61	
	Maternal age (years)	27·6		5·0	28·6		3·4	27·4		6·0
	Parity	2·8		1·6	2·8		1·0	2·8		1·9
	Paternal age (years)	31·7		6·3	32·8		4·0	31·5		7·7
	Maternal education									
	No schooling	34·5	396		32·1	161		35·1	235	
	Paternal education									
	No schooling	13·8	184		19·3	98		12·5	86	
Household characteristics	Food insecurity scale (score 0–8)	3·1		2·6	3·2		1·8	3·1		3·2
	Household size	7·4		3·3	6·7		1·8	7·5		4·2
	WASH quintile	2·0		1·5	2·1		0·9	2·0		1·8
	Wealth quintile	2·4		1·3	2·5		0·7	2·4		1·7

WASH, water, sanitation and hygiene.

* Food insecurity scale is out of 8 with increasing scores representing increasing food insecurity. Higher WASH and wealth quintiles represent cleaner and wealthier households, respectively.

**Table 2.** Health and nutrition characteristics of preschool-age children living in Uttar Pradesh^*^

		State survey sample			Urban state survey sample			Rural state survey sample		
		Mean or %	*n*	sd	Mean or %	*n*	sd	Mean or %	*n*	sd
Dietary diversity										
	*n*	1238			582			656		
	Dietary diversity score (score 0–7)	2·5		1·3	2·5		0·89	2·6		1·6
	Starches	86·8	1059		84·2	487		87·3	572	
	Legumes and nuts	37·6	458		29·9	210		39·3	248	
	Dairy products	65·8	800		64·1	376		66·1	424	
	Flesh foods	4·8	78		7·4	51		4·2	27	
	Eggs	4·4	58		6·8	34		3·9	24	
	Vitamin A-rich fruits and vegetables	28·9	349		30·0	172		28·6	177	
	Other fruits and vegetables	26·7	308		24·5	143		27·2	165	
Anthropometry										
	*n*	1225			578			647		
	Length-for-age z-score	−1·85		1·6	−1·64		1·7	−1·90		1·6
	Stunted (< –2 sd)	47·7	570		44·0	255		48·6	315	
	Severely stunted (< –3 sd)	20·6	259		15·9	117		21·7	142	
	Weight-for-length z-score	−0·55		1·0	−0·55		1·0	−0·55		10
	Wasted (< –2 sd)	6·7	86		4·6	40		7·1	46	
	Severely wasted (< –3 sd)	1·2	16		0·9	6		1·3	10	
	Weight-for-age z-score	−1·47		1·4	−1·31		1·4	−1·50		1·3
	Underweight (< –2 sd)	32·5	396		26·6	180		33·8	216	
	Severely underweight (< –3 sd)	10·8	129		8·0	58		11·4	71	
Anaemia										
	*n*	944			427			517		
	Any (Hb < 11 g/dL)	56·0	522		59·9	240		55·2	282	
	Mild (10 g/dL ≤ Hb < 11 g/dL)	25·6	222		22·8	93		26·1	129	
	Moderate (7 g/dL ≤ Hb < 10 g/dL)	27·9	274		34·0	134		26·6	140	
	Severe (Hb < 7 g/dL)	2·5	26		3·0	13		2·4	13	
	Mean (sd) Hb (g/dL)	10·6		1·7	10·4		1·1	10·6		2·1
Ferritin (adjusted for CRP and AGP using BRINDA method)										
	*n*	757			343			414		
	% low ferritin (< 12 µg/L)	64·3	492		71·4	229		62·7	263	
	Geometric mean (95 % C) ferritin (µg/L)	8·5	7·5, 9·7		6·8	6·0, 7·7		9·0	7·9, 10·2	
Ferritin (unadjusted for inflammation)										
	*n*	757			343			414		
	% low ferritin (< 12 µg/L)	52·7	407		63·0	198		50·3	209	
	Geometric mean (95 % C) ferritin (µg/L)	11·4	10·0, 13·1		8·6	7·7, 9·6		12·2	10·7, 13·9	
%Fe deficiency anaemia (adjusted ferritin < 12 µg/l and hb < 11 g/dL)										
	*n*	750			338			412		
		44·6	332		51·7	153		43·0	179	
Vitamin B_12_										
	*n*	681			304			377		
	% deficiency (< 150 pmol/L)	12·3	81		9·1	31		13·0	50	
	Geometric mean (95 % CI) vitamin B_12_ (pmol/L)	252·8	241·0, 265·2		261·5	248·8, 274·8		250·9	223·0, 275·5	
RBC folate										
	*n*	779			352			427		
	% deficiency (< 305 nmol/L)	7·6	59		8·8	31		6·6	28	
	Geometric mean (95 % CI) RBC folate (nmol/L)	685·7	615·2, 764·2		645·5	578·2, 727·8		692·3	629·2, 780·6	
Zn (adjusted for CRP and AGP using BRINDA method, and for time of last meal and time of processing)										
	*n*	857			391			466		
	% deficiency (< 9·9 µmol/L)	29·3	274		27·1	120		29·8	154	
	Geometric mean (95 % CI) Zn (µmol/L)	11·9	11·1, 12·7		11·7	11·0, 12·4		11·9	11·0, 12·8	
Zn (unadjusted)										
	*n*	857			391			466		
	% deficiency (< 9·9 µmol/L)	37·6	345		35·5	152		38·1	193	
	Geometric mean (95 % CI) Zn (µmol/L)	11·1	10·4, 11·8		11·0	10·4, 11·6		11·1	10·3, 11·9	
Retinol (adjusted for CRP and AGP using BRINDA method)										
	*n*	778			354			424		
	% deficiency (< 0·7 µmol/L)	51·3	387		44·4	163		52·8	224	
	Geometric mean (95 % CI) retinol (µmol/L)	0·60	0·56, 0·64		0·64	0·59, 0·70		0·59	0·54, 0·64	
Retinol (unadjusted for inflammation)										
	*n*	778			354			424		
	% deficiency (< 0·7 µmol/L)	55·5	426		47·7	181		57·2	245	
	Geometric mean (95 % CI) retinol (µmol/L)	0·60	0·55, 0·64		0·64	0·57, 0·69		0·59	0·54, 0·64	
C-reactive protein										
	*n*	757			343			414		
	% elevated CRP (> 5 mg/L)	9·6	68		7·7	29		9·8	40	
	Geometric mean (95 % CI) CRP (mg/L)	0·45	0·37, 0·56		0·34	0·21, 0·55		0·48	0·38, 0·61	
*α*1-acid glycoprotein										
	*n*	755			343			414		
	% elevated AGP (> 1 g/L)	40·2	316		35·9	137		41·2	179	
	Geometric mean (95 % CI) AGP (g/L)	0·93	0·87, 0·98		0·89	0·81, 0·98		0·93	0·88, 0·99	
Malaria										
	*n*	942			426			516		
		0·43	3		0	0		0·39	3	

AGP, *α*1-acid glycoprotein; CRP, C-reactive protein.

* Morbidity measured as any episodes of fever, cough and diarrhoea in the past week.

**Table 3:** Health and nutrition characteristics of mothers of reproductive age living in Uttar Pradesh

		State survey sample			Urban state survey sample			Rural state survey sample		
		Mean or %	*n*	sd	Mean or %	*n*	sd	Mean or %	*n*	sd
Dietary diversity										
	*n*	1238			582			656		
	Dietary diversity score (score 0–9)	2·8		1·2	2·8		0·82	2·8		1·4
	Starches	99·8	1236		99·9	581		99·8	655	
	Legumes and nuts	44·6	561		38·5	269		45·9	292	
	Dairy products	42·7	522		35·7	234		44·2	288	
	Meat and fish	7·1	98		7·6	54		7·1	44	
	Eggs	5·1	75		9·8	52		4·1	23	
	Organ meat	2·4	41		6·0	30		1·7	11	
	Vitamin A-rich fruits and vegetables	10·5	129		13·5	69		9·8	60	
	Dark green leafy vegetables	33·3	425		39·1	217		32·0	208	
	Other fruits and vegetables	30·5	382		27·1	192		31·2	190	
Anthropometry										
	*n*	1238			582			656		
	Mean BMI	21·6		4·4	22·5		2·7	21·4		5·4
	≥ 30	4·0	50		5·8	29		3·7	21	
	25–29·9	14·8	196		20·2	111		13·6	85	
	18·5–24·9	55·3	712		60·3	355		54·2	357	
	< 18·5	25·9	280		13·7	87		28·6	193	
Anaemia										
	*n*	1092			495			597		
	Any (Hb < 12 g/dL)	36·4	379		33·1	159		37·1	220	
	Mild (11 g/dL ≤ Hb < 12 g/dL)	18·7	190		13·0	74		20·0	116	
	Moderate (8 g/dL ≤ Hb < 11 g/dL)	15·1	167		17·7	76		14·6	91	
	Severe (Hb < 8 g/dL)	2·5	22		2·4	9		2·5	13	
	Mean (sd) Hb (g/dL)	12·3		1·8	12·4		1·1	12·3		2·2
Ferritin (adjusted for CRP and AGP using BRINDA method)										
	*n*	1036			471			565		
	% low ferritin (< 15 µg/L)	51·0	520		51·6	238		50·9	282	
	Geometric mean (95 % CI) ferritin (µg/L)	15·9	13·9, 18·2		14·4	12·6, 16·4		16·2	13·9, 19·0	
Ferritin (unadjusted for inflammation)										
	*n*	1036			471			565		
	% low ferritin (< 15 µg/L)	39·7	411		41·2	190		39·4	221	
	Geometric mean (95 % CI) ferritin (µg/L)	20·0	17·4, 22·9		18·4	16·1, 21·0		20·3	17·4, 23·7	
%Fe deficiency anaemia (adjusted ferritin < 15 µg/L and hb < 12 g/dL)										
	*n*	1034			469			565		
		26·7	262		24·3	109		27·2	153	
Vitamin B^12^										
	*n*	1009			457			552		
	% deficiency (< 150 pmol/L)	17·2	174		19·6	80		16·7	94	
	Geometric mean (95 % CI) vitamin B_12_ (pmol/L)	245·0	223·4, 268·7		232·0	213·7, 251·8		247·9	223·0, 275·5	
Red blood cell folate										
	*n*	1034			466			568		
	% deficiency (< 305 nmol/L)	16·3	169		17·4	81		15·5	88	
	Geometric mean (95 % CI) RBC folate (nmol/L)	554·5	485·4, 633·5		507·8	454·9, 566·8		566·8	487·8, 652	
Zn (unadjusted)										
	*n*	1041			470			571		
	% deficiency (< 10·7 µmol/L)	50·0	559		48·5	254		50·4	305	
	Geometric mean (95 % CI) Zn (µmol/L)	10·6	9·8, 11·4		10·7	10·2, 11·3		10·5	9·7, 11·5	
Retinol (unadjusted for inflammation)										
	*n*	884			404			480		
	% deficiency (< 0·7 µmol/L)	40·8	351		32·3	144		42·6	207	
	Geometric mean (95 % CI) retinol (µmol/L)	0·73	0·68, 0·79		0·81	0·76, 0·86		0·71	0·65, 0·78	
C-reactive protein										
	*n*	1036			471			565		
	% elevated CRP (> 5 mg/L)	8·5	96		10·3	73		8·1	43	
	Geometric mean (95 % CI) CRP (mg/L)	0·59	0·46, 0·75		0·67	0·47, 0·95		0·57	0·45, 0·74	
*α*1-acid glycoprotein										
	*n*	1036			471			565		
	% elevated AGP (> 1 g/L)	29·9	335		31·1	161		29·7	174	
	Geometric mean (95 % CI) AGP (g/L)	0·82	0·77, 0·87		0·83	0·77, 0·90		0·82	0·77, 0·87	
*β* thalassaemia trait (HbA2 > 3·2)										
	*n*	744			353			391		
		3·0	19		3·4	10		2·9	9	
Malaria										
	*n*	1091			495			596		
		0	0		0	0		0	0	

AGP, *α*1-acid glycoprotein; CRP, C-reactive protein.

The fraction of anaemia attributable to low Fe was 37·8 % in PSC and 15·0 % in MRA. Other population attributable fraction percentages were below 15 % (Tables 4 and 5). In PSC, the correlation matrix indicated significant correlations between Hb and ferritin, RBC folate, vitamin B_12_, age and birth weight (see online supplementary material, Supplemental Table 1). In MRA, correlations were significant between Hb and ferritin, retinol, RBC folate and food insecurity (see online supplementary material, Supplemental Table 2).

**Table 4:** Multivariable analysis of the risk factors for anaemia and their population attributable fraction in preschool-age children^*^

	Anaemia PR	95 % CI	*P*	PAF (%)
Fe deficiency (*n* 743)				
Serum ferritin ≥ 12 µg/L	ref			
Serum ferritin < 12 µg/L	1·84	1·55, 2·19	<0·001	37·8
Vitamin B_12_ (*n* 670)				
Serum vitamin B_12_ ≥ 150 pmol/L	ref			
Serum vitamin B_12_ < 150 pmol/L	0·89	0·73, 1·09	0·264	0·7
Folate (*n* 772)				
RBC folate ≥ 305 nmol/L	ref			
RBC folate < 305 nmol/L	0·63	0·44, 0·90	0·011	6·5
Zn (*n* 842)				
Serum Zn ≥ 9·9 µmol/L	ref			
Serum Zn < 9·9 µmol/L	1·00	0·89, 1·14	0·939	0·4
Vitamin A (*n* 768)				
Serum retinol ≥ 0·7 µmol/L	ref			
Serum retinol < 0·7 µmol/L	1·07	0·95, 1·21	0·2446	9·9
Inflammation (CRP *n* 743; AGP *n* 741)				
C-reactive protein ≤ 5 mg/L	ref			
C-reactive protein > 5 mg/L	1·09	0·91, 1·31	0·357	2·9
* α*1-acid glycoprotein ≤ 1 g/L	ref			
* α*1-acid glycoprotein > 1 g/L	1·07	0·95, 1·20	0·285	7·2
Setting (*n* 937)				
Urban	ref			
Rural	1·00	0·89, 1·11	0·958	3·8
Any food insecurity (*n* 937)				
No	ref			
Yes	1·06	0·93, 1·21	0·387	6·4
Dietary diversity (*n* 937)				
≥ 4 food groups	ref			
< 4 food groups	1·08	0·93, 1·24	0·310	10·2
WASH (*n* 937)				
First quintile	ref			
Second quintile	1·00	0·85, 1·19	0·956	10·4
Third quintile	0·94	0·80, 1·19	0·514	
Fourth quintile	0·83	0·68, 1·01	0·061	
Fifth quintile	0·98	0·83, 1·16	0·832	

PR, prevalence ratio; PAF, population attributable fraction; ref, reference.

* Analyses adjust for child age. Serum ferritin and retinol were adjusted for inflammation using BRINDA method. Serum Zn was adjusted for inflammation using BRINDA method, and for time of last meal and time of processing.

**Table 5:** Multivariable analysis of the risk factors for anaemia and their population attributable fraction in mothers of reproductive age^*^

	Anaemia PR	95 % CI	*P*	PAF (%)
Fe deficiency (*n* 1034)				
Serum ferritin ≥ 15 µg/L	ref			
Serum ferritin < 15 µg/L	2·90	2·36, 3·56	< 0·001	15·0
Vitamin B_12_ (*n* 1007)				
Serum vitamin B_12_ ≥ 150 pmol/L	ref			
Serum vitamin B_12_ < 150 pmol/L	1·08	0·86, 1·34	0·521	1·9
Folate (*n* 1034)				
RBC folate ≥ 305 nmol/L	ref			
RBC folate < 305 nmol/L	0·62	0·46, 0·84	0·001	10·2
Zn (*n* 1038)				
Serum Zn ≥ 10·7 µmol/L	ref			
Serum Zn < 10·7 µmol/L	1·19	1·00, 1·42	0·047	10·4
Vitamin A (*n* 883)				
Serum retinol ≥ 0·7 µmol/L	ref			
Serum retinol < 0·7 µmol/L	1·29	1·07, 1·55	0·008	14·5
Inflammation (*n* 1034)				
C-reactive protein ≤ 5 mg/L	ref			
C-reactive protein > 5 mg/L	1·18	0·91, 1·54	0·212	2·5
*α*1-acid glycoprotein ≤ 1 g/L	ref			
*α*1-acid glycoprotein > 1 g/L	0·99	0·83, 1·19	0·922	0·4
Setting (*n* 1092)				
Urban	ref			
Rural	1·15	0·97, 1·35	0·104	0
Any food insecurity (*n* 1092)				
No	ref			
Yes	1·03	0·86, 1·24	0·734	0
Dietary diversity (*n* 1092)				
≥ 5 food groups	ref			
< 5 food groups	1·04	0·76, 1·44	0·789	0
WASH (*n* 1092)				
First quintile	ref			
Second quintile	1·18	0·92, 1·52	0·200	12·2
Third quintile	1·00	0·77, 1·32	0·978	
Fourth quintile	1·16	0·90, 1·51	0·253	
Fifth quintile	0·95	0·73, 1·24	0·707	

PR, prevalence ratio; PAF, population attributable fraction; ref, reference.

* Serum ferritin was adjusted for inflammation using BRINDA method. Serum Zn was adjusted for time of last meal and time of processing.

**Fig. 1:**
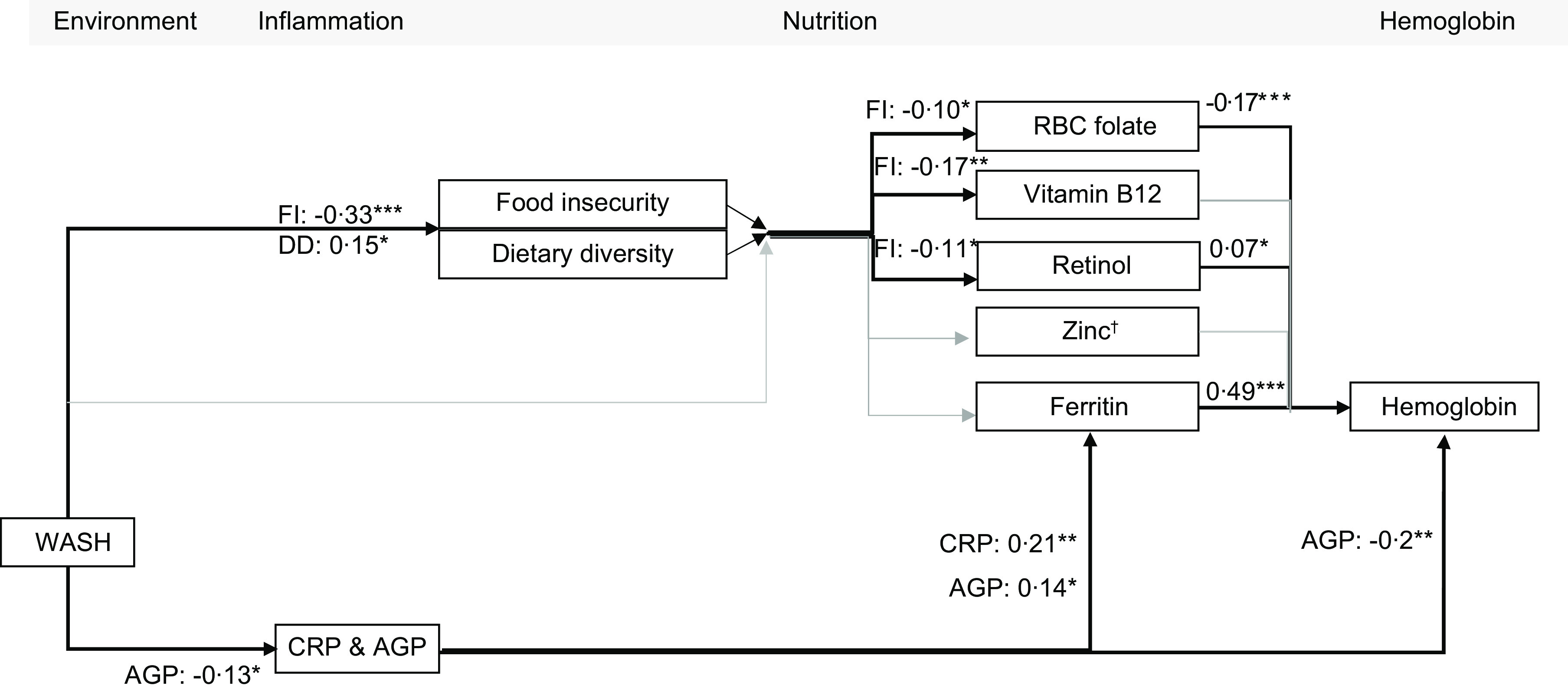
Path model of the predictors of Hb concentration in preschool-age children living in Uttar Pradesh. *Note*: Results are presented as standardised estimates, adjusted for age in months. The model utilised log transformed Zn, folate, vitamin B_12_, retinol, CRP and AGP. WASH is represented in quintiles; all other variables are continuous scores or concentrations. Thin grey arrows represent non-significant associations; thick black arrows represent significant associations. Model fit statistics: RMSEA = 0·057; CFI = 0·960; TLI = 0·836; SRMR = 0·0034. Direct associations between inflammation biomarkers and nutritional biomarkers were estimated, but not all are presented; significant direct associations were also found between AGP and Zn (*β* = –0·15*), AGP and vitamin B_12_ (*β* = 0·02*), and CRP and vitamin B_12_ (*β* = –0·01*). AGP, *α*-1 acid glycoprotein; CRP, C-reactive protein; DD, dietary diversity score; FI, food insecurity score; WASH, water, sanitation and hygiene; RMSEA, root mean square error of approximation; CFI, comparative fit index; TLI, Tucker Lewis index; SRMR, standardised root mean squared residual. † Zn adjusted for time of processing and fasting. *P < 0·05. **P < 0·01. ***P < 0·001

In PSC, the path model indicated that RBC folate (standardised *β* (*β*) = –0·17, *P* < 0·001), retinol (*β* = 0·07, *P* = 0·034), ferritin (*β* = 0·49, *P* < 0·001) and AGP (*β* = 0·20, *P* = 0·002) were significantly directly associated with Hb concentrations (Fig. 1, see online supplementary material, Supplemental Table 3). CRP was indirectly associated with Hb through ferritin (see online supplementary material, Supplemental Tables 3 and 4). Both CRP (*β* = 0·21, *P* = 0·002) and AGP (*β* = 0·14, *P* = 0·016) were significantly directly associated with ferritin. Food insecurity (increasing insecurity) was negatively associated with RBC folate, vitamin B_12_ and retinol concentrations. WASH quintile (increasing cleanliness) was negatively associated with food insecurity (*β* = –0·33, *P* < 0·001) and AGP (*β* = –0·13, *P* = 0·029) and positively associated with dietary diversity (*β* = 0·15, *P* = 0·012) (Fig. 1).

**Fig. 2:**
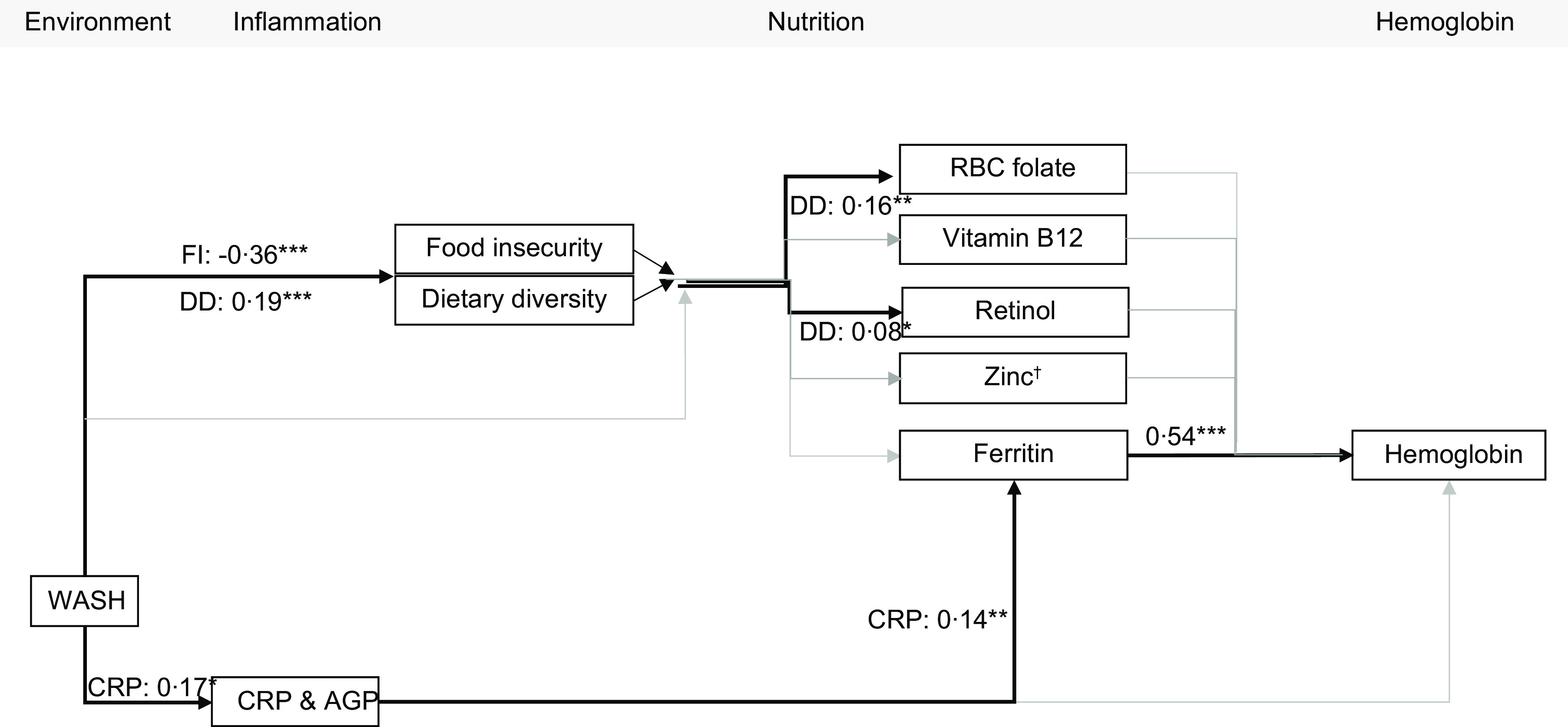
Path model of the predictors of Hb concentration in mothers of reproductive age living in Uttar Pradesh. *Note*: Results are presented as standardised estimates. The model utilised log transformed Zn, folate, vitamin B_12_, retinol, CRP and AGP. WASH is represented in quintiles; all other variables are continuous scores or concentrations. Thin grey arrows represent non-significant associations; thick black arrows represent significant associations. Model fit statistics: RMSEA = 0·058; CFI = 0·926; TLI = 0·773; SRMR = 0·036. Direct associations between inflammation biomarkers and nutritional biomarkers were estimated, but not all are presented; significant associations were also found between AGP and vitamin B_12_ (*β* = 0·11*) and CRP and folate (*β* = 0·09**). AGP, *α*-1 acid glycoprotein; CRP, C-reactive protein; DD, dietary diversity score; FI, food insecurity score; WASH, water, sanitation and hygiene; RMSEA, root mean square error of approximation; CFI, comparative fit index; TLI, Tucker Lewis index; SRMR, standardised root mean squared residual. † Zn adjusted for time of processing and fasting. *P < 0·05. **P < 0·01. ***P < 0·001

In MRA, the path model indicated that only ferritin (*β* = 0·54, *P* < 0·001) was significantly directly associated with Hb concentration (Fig. 2, see online supplementary material, Supplemental Table 5). CRP was indirectly associated with Hb through ferritin (see online supplementary material, Supplemental Tables 5 and 6). CRP was significantly associated with ferritin (*β* = 0·14, *P* = 0·006), but AGP was not. Dietary diversity was associated with RBC folate and retinol concentrations. WASH was negatively associated with food insecurity (*β* = –0·36, *P* < 0·001) and positively associated with dietary diversity (*β* = 0·19, *P* < 0·001) and CRP (*β* = 0·17, *P* = 0·010) (Fig. 2).

For the additional analysis including *β*-thalassaemia in the MRA model, the path model indicated that both ferritin (*β* = 0·58, *P* < 0·001) and *β*-thalassaemia (*β* = –0·038, *P* = 0·019) were significantly directly associated with Hb concentration. There were no meaningful differences in the estimates between models with and without *β*-thalassaemia trait (see online supplementary material, Supplemental Tables 7–8).

No meaningful differences were found in the estimates between models using full information maximum likelihood estimation and models using maximum likelihood estimation in both PSC and MRA (see online supplementary material, Supplemental Tables 9–12). Basic descriptive variables (i.e. age, child sex and Hb concentration) were comparable across those with missing and non-missing data. In PSC and MRA models, no differences between rural and urban settings were found for any of the pathways.

## Discussion

In mothers and children in Uttar Pradesh, ferritin, a measure of body Fe stores, appears to be the single most important correlate of Hb concentration. This is consistent with the literature on the importance of ferritin in the aetiology of anaemia^(6,44–46)^. Also consistent^(5)^ is the existence of other determinants of anaemia, namely inflammation, folate and retinol. The path analysis illustrates how distal factors, such as dietary diversity, food insecurity and WASH, work through influences on nutritional status and inflammation indicators. Many Fe interventions (supplementation among others) are effective in increasing Hb concentration and reducing anaemia, but the magnitude of that reduction is often not as large as anticipated from the prevalence of Fe deficiency^(47)^. The extent to which this may be due at least in part to the complex interrelationship among nutritional status, inflammation and Hb concentration illustrated by our path analysis has not been adequately studied.

Those concerns notwithstanding, approximately 78 % of children and 74 % of mothers with anaemia, had Fe deficiency (as defined by low ferritin). These statistics and the path analysis estimates suggest that Fe status is the most important problem in this context, which urgently needs to be addressed. In both women and child models, ferritin was the strongest statistical predictor of Hb concentration. These findings complement other studies in South Asia where malaria prevalence was also low. Results from Vietnam and India indicate significant increased odds of anaemia in women and children with Fe deficiency^(6,7,48)^. The study in Northern Vietnam found that 11 % of anaemia among women of reproductive age was attributable to insufficient Fe stores^(6)^.

In our study, a negative association was found between folate and Hb concentration in children. While others have found contrasting results of positive associations between folate and Hb^(49)^, some have found similar inverse associations in low- and middle-income school-age children living in Bogota, Colombia^(50)^ and in elderly Americans^(51)^. In Colombia, few children had low folate status, likely because wheat flour is fortified with folic acid in the country^(52)^. In the USA, high serum folate was associated with anaemia only in older adults with low vitamin B_12_^(51)^. The negative association between folate and Hb in populations with low folate deficiency, as is the case in our study (only 8·7 % of PSC and 18·3 % of MRA were folate deficient), and particularly among individuals with low vitamin B_12_ status is not well understood^(53,54)^. Some have stipulated that a high folate intake could create a competitive reduction in Fe absorption or metabolism, which would result in less Hb synthesis^(55–57)^. From another perspective, individuals with elevated folate may be consuming foods high in folate, which are also high in phytates, ultimately reducing Fe absorption, a hypothesis that we could not test in this analysis. Further, those consuming high cereal diet, resulting in high folate, may also have lower intake of high-quality protein necessary for Hb production^(58)^.

After micronutrient status, the strongest predictor of Hb is inflammation, a marker of infection, which can reduce appetite, use nutrient resources in fighting infections and reduce absorption^(59,60)^. These findings are consistent with a pooled analysis of sixteen national surveys in PSC and ten surveys in WRA, in which inflammation was repeatedly found to be significantly associated with anaemia, particularly in contexts with a high burden of infection. The association between inflammation and Hb, independently of Fe, has been demonstrated in studies in young Indian children^(7)^, Danish blood donors^(61)^ and Sierra Leonean children and women^(10)^. Inflammation has also been shown to be indirectly associated with Hb by acting on hepcidin, an Fe regulating peptide hormone, causing the down-regulation of Fe absorption and erythropoiesis^(62)^.

Genetic Hb disorders have been identified as important contributors to anaemia. In a study of WRA in Cambodia, Hb E homozygous disorder was one of the strongest predictors of Hb, even before Fe status; heterozygous Hb E and Constant Spring traits were also important statistical predictors of Hb concentration^(63)^. Pasricha and colleagues also showed a significant association between *β*-thalassaemia minor and Hb in Indian children^(7)^. Our results complement these findings by indicating a direct association between *β*-thalassaemia and Hb concentration in our population of women.

The population studied showed a high prevalence of concurrent Fe deficiency and anaemia; therefore, the statistical contributions of nutritional predictors of anaemia are likely generalisable to populations with similarly high prevalence of Fe deficiency and anaemia but needs to be explored further in populations where Fe deficiency may not be associated with such a high proportion of anaemia, such as settings with malaria or high proportions of genetic abnormalities. Our study captures low prevalence of acute inflammation (CRP) and higher prevalence of chronic inflammation (AGP), which could be reflective of seasonality. October to December is the beginning of winter, and the post-rainy season when episodes of diarrhoea and incidence of malaria are typically lower compared to the summer months^(64,65)^. Results are likely to be different in seasons with higher incidence of infectious disease. The implications of this seasonal pattern of inflammation for the potential for impact of Fe interventions should also be explored.

The cross-sectional nature of the data does not allow us to establish the associations as causal. Further, path analysis could be biased when applied to cross-sectional data, particularly when the effects of mediation unfold over time^(66)^. Longitudinal data should be used to confirm these findings and estimates of association. Ten of the twenty-five districts were not randomly selected because of the dual purpose of this survey, serving as a state survey and a baseline evaluation to a double fortified salt programme. However, we expect that results are representative of the state because of the geographical spread of selected districts, design and rigorous sampling methods within each district. Our sample of children unexpectedly included fewer girls than boys, which could have introduced some bias in our results given that certain blood disorders, such as glucose-6-phosphate dehydrogenase deficiency, are more common in males than females^(67)^. Although we tested extensively for nutritional deficiencies, inflammatory biomarkers and more distal social and environmental factors which may affect anaemia, we were not able to test for all possible predictors. Importantly, we did not conduct egg counts of intestinal parasites, nor did we test for genetic Hb disorders other than *β*-thalassaemia. The use of path analysis allowed for a more complex view of the predictors of anaemia in this population, accounting for both direct and indirect associations between predictors, and allowing for comparability in magnitudes of associations between predictors and between settings.

The associations observed in this analysis begin to illustrate the complex aetiology of anaemia and the potential interrelationship between several nutritional and immune function-related causes, as well as their relations with more distal determinants such as WASH and food security. In both women and children, ferritin, a marker reflective of Fe stores, is still the single strongest correlate of Hb concentration, but other nutritional deficiencies are also important statistical predictors. The nature of these relationships, particularly with infection and inflammation, however, is not straightforward, and the extent to which Fe and other nutritional interventions can be fully effective to address anaemia is not clear. Even for the proportion of anaemia that, according to our model, is attributable to Fe deficiency, other causes may inhibit a response to Fe interventions if not simultaneously addressed. Our models suggest that interventions using experimental study designs that simultaneously address multiple nutritional predictors as well as control of infection and inflammation will have greater potential to reduce the prevalence of anaemia. More complex models and comprehensive measurements, as introduced in this study, are needed to more fully understand the aetiology of anaemia and populations’ potential responses to interventions, so as to design cost-effective solutions to an important public health problem.

## Supporting information

Larson et al. supplementary material Larson et al. supplementary material

## Supplementary material

For supplementary material accompanying this paper visit https://doi.org/10.1017/S1368980024000028
