# Supplementary material for: Predictors of anaemia in mothers and children in Uttar Pradesh, India
Source: Public Health Nutr. 2024 Jan 8;27(1):e30. doi: 10.1017/S1368980024000028 (PMC10830375; doi:10.1017/S1368980024000028)
Supplement: Larson et al. supplementary material [file S1368980024000028sup001.docx]

Supplemental Table 1: Correlation coefficients (p-value) in children 6-59 months living in Uttar Pradesh (N=507)^1^

|  | | | | | | | | | | | | |  |  |  |  |
| --- | --- | --- | --- | --- | --- | --- | --- | --- | --- | --- | --- | --- | --- | --- | --- | --- |
|  | Hemoglobin (g/dL) | Ln Ferritin (ug/L)^1^ | Ln retinol (umol/L) | RBC folate | Ln vitamin B12 (pmol/L) | Ln zinc (umol/L) | Ln CRP (mg/L) | Ln AGP (g/L) | WASH quintile | Dietary diversity score | Food insecurity score | Low birth weight | Age in months | Male | Wealth quintile | Rural |
| Ln Ferritin (ug/L) | 0.52 (<0.001) |  |  |  |  |  |  |  |  |  |  |  |  |  |  |  |
| Ln retinol (umol/L) | 0.04 (0.224) | -0.02 (0.661) |  |  |  |  |  |  |  |  |  |  |  |  |  |  |
| RBC folate | -0.20 (<0.001) | -0.09 (0.020) | 0.08 (0.029) |  |  |  |  |  |  |  |  |  |  |  |  |  |
| Ln vitamin B12 (pmol/L) | -0.15 (<0.001) | -0.14 (<0.001) | 0.07 (0.065) | 0.11 (0.007) |  |  |  |  |  |  |  |  |  |  |  |  |
| Ln zinc (umol/L) | 0.01 (0.784) | 0.02 (0.548) | 0.10 (0.004) | 0.03 (0.355) | -0.06 (0.154) |  |  |  |  |  |  |  |  |  |  |  |
| Ln CRP (mg/L) | 0.02 (0.629) | 0.06 (0.079) | -0.04 (0.228) | 0.03 (0.448) | -0.02 (0.596) | 0.01 (0.886) |  |  |  |  |  |  |  |  |  |  |
| Ln AGP (g/L) | -0.07 (0.051) | 0.06 (0.089) | -0.04 (0.303) | 0.05 (0.208) | 0.14 (<0.001) | -0.05 (0.200) | 0.50 (<0.001) |  |  |  |  |  |  |  |  |  |
| WASH quintile | 0.01 (0.964) | -0.03 (0.422) | 0.03 (0.410) | 0.03 (0.405) | 0.03 (0.479) | 0.06 (0.065) | -0.07 (0.053) | -0.10 (0.005) |  |  |  |  |  |  |  |  |
| Dietary diversity score | 0.06 (0.086) | -0.02 (0.531) | 0.11 (0.003) | 0.01 (0.742) | 0.08 (0.035) | 0.07 (0.051) | -0.09 (0.016) | -0.02 (0.586) | 0.09 (0.001) |  |  |  |  |  |  |  |
| Food insecurity score | -0.01 (0.694) | 0.01 (0.756) | -0.04 (0.264) | -0.08 (0.022) | -0.12 (0.003) | -0.08 (0.022) | 0 (0.967) | 0.02 (0.654) | -0.04 (<0.001) | -0.13 (<0.001) |  |  |  |  |  |  |
| Low birth weight | -0.11 (0.015) | -0.07 (0.148) | 0.04 (0.442) | 0.06 (0.238) | 0.04 (0.133) | -0.07 (0.121) | 0 (0.951) | -0.03 (0.489) | -0.04 (0.360) | -0.02 (0.558) | 0.06 (0.138) |  |  |  |  |  |
| Age in months | 0.28 (<0.001) | 0.19 (<0.001) | 0.01 (0.827) | -0.16 (<0.001) | 0.01 (0.700) | -0.01 (0.800) | -0.03 (0.440) | -0.01 (0.877) | -0.11 (<0.001) | 0.22 (<0.001) | 0.04 (0.123) | 0.08 (0.034) |  |  |  |  |
| Male | -0.05 (0.153) | -0.06 (0.083) | 0.01 (0.756) | 0.05 (0.169) | 0 (0.961) | 0.07 (0.040) | -0.04 (0.244) | 0.03 (0.467) | 0.01 (0.646) | 0.01 (0.608) | -0.10 (<0.001) | 0.01 (0.790) | -0.01 (0.700) |  |  |  |
| Wealth quintile | 0.03 (0.303) | -0.04 (0.285) | 0.10 (0.007) | 0.03 (0.399) | 0.06 (0.132) | 0.07 (0.033) | -0.05 (0.190) | -0.13 (<0.001) | 0.53 (<0.001) | 0.20 (<0.001) | -0.20 (<0.001) | -0.01 (0.740) | -0.12 (<0.001) | -0.01 (0.965) |  |  |
| Rural | 0.04 (0.281) | 0.14 (<0.001) | -0.04 (0.252) | 0.04 (0.210) | -0.04 (0.374) | 0.01 (0.694) | 0.07 (0.060) | 0.04 (0.276) | -0.02 (0.536) | 0.03 (0.300) | -0.01 (0.795) | -0.01 (0.916) | 0.05 (0.100) | 0.03 (0.192) | -0.05 (0.113) |  |
| Vitamin A supplementation in the past 6 months | -0.05 (0.100) | -0.08 (0.039) | 0.03 (0.344) | 0.10 (0.004) | 0.01 (0.894) | -0.05 (0.172) | 0.01 (0.772) | 0.02 (0.623) | 0.074 (0.010) | 0.10 (<0.001) | 0.02 (0.382) | 0.02 (0.531) | -0.04 (0.142) | -0.04 (0.135) | 0.08 (0.003) | 0.01 (0.739) |
|  |  |  |  |  |  |  |  |  |  |  |  |  |  |  |  |  |
| ^1^Food insecurity scale is out of 8 with increasing scores representing increasing food insecurity. Higher WASH and wealth quintiles represent cleaner and wealthier households, respectively. Ferritin and retinol were adjusted for CRP and AGP using BRINDA method. sTfR was adjusted for AGP using BRINDA method. Zinc was adjusted for CRP and AGP, time of processing, and fasting level. | | | | | | | | | | | | | | | | |

Supplemental Table 2: Correlation coefficients (p-value) in non-pregnant mothers of reproductive age living in Uttar Pradesh (N=798)^1^

|  | Hemoglobin (g/dL) | Ln Ferritin (ug/L)^1^ | Ln sTfR (mg/L) | Ln retinol (umol/L) | RBC folate | Ln vitamin B12 (pmol/L) | Ln zinc (umol/L) | Ln CRP (mg/L) | Ln AGP (g/L) | β thalassemia | WASH quintile | Dietary diversity score | Food insecurity score | Age in years | Wealth quintile |
| --- | --- | --- | --- | --- | --- | --- | --- | --- | --- | --- | --- | --- | --- | --- | --- |
| Ln Ferritin (ug/L) | 0.53 (<0.001) |  |  |  |  |  |  |  |  |  |  |  |  |  |  |
| Ln sTfR (mg/L) | -0.34 (<0.001) | -0.38 (<0.001) |  |  |  |  |  |  |  |  |  |  |  |  |  |
| Ln retinol (umol/L) | 0.07 (0.049) | 0.07 (0.042) | 0.04 (0.226) |  |  |  |  |  |  |  |  |  |  |  |  |
| RBC folate | -0.12 (<0.001) | -0.09 (0.003) | 0.13 (<0.001) | 0.10 (0.003) |  |  |  |  |  |  |  |  |  |  |  |
| Ln vitamin B12 (pmol/L) | 0.05 (0.088) | -0.02 (0.492) | 0.02 (0.493) | -0.04 (0.310) | 0.17 (<0.001) |  |  |  |  |  |  |  |  |  |  |
| Ln zinc (umol/L) | 0.05 (0.086) | 0.02 (0.556) | 0.08 (0.014) | 0.09 (0.006) | 0.12 (<0.001) | 0.02 (0.476) |  |  |  |  |  |  |  |  |  |
| Ln CRP (mg/L) | 0.03 (0.266) | 0.02 (0.541) | 0.28 (<0.001) | 0.03 (0.440) | 0.09 (0.007) | 0.06 (0.072) | 0.01 (0.791) |  |  |  |  |  |  |  |  |
| Ln AGP (g/L) | 0.04 (0.158) | 0 (0.879) | 0.01 (0.763) | -0.05 (0.120) | 0.03 (0.368) | 0.11 (<0.001) | -0.04 (0.172) | 0.47 (<0.001) |  |  |  |  |  |  |  |
| β thalassemia | -0.01 (0.708) | -0.02 (0.571) | -0.02 (0.547) | 0.01 (0.854) | 0.04 (0.270) | -0.07 (0.102) | -0.03 (0.416) | 0.14 (<0.001) | 0.07 (0.090) |  |  |  |  |  |  |
| WASH quintile | 0.05 (0.116) | 0.04 (0.249) | 0.08 (0.007) | 0.04 (0.208) | 0.04 (0.239) | 0.06 (0.053) | 0.01 (0.794) | 0.12 (<0.001) | 0.041 (0.196) | -0.01 (0.914) |  |  |  |  |  |
| Dietary diversity score | 0.04 (0.204) | 0.02 (0.581) | 0.01 (0.770) | 0.07 (0.047) | 0.13 (<0.001) | 0.08 (0.012) | -0.05 (0.098) | 0.03 (0.287) | 0.05 (0.090) | 0.07 (0.065) | 0.17 (<0.001) |  |  |  |  |
| Food insecurity score | -0.08 (0.011) | -0.06 (0.042) | -0.03 (0.276) | -0.05 (0.157) | -0.08 (0.007) | -0.03 (0.393) | -0.02 (0.484) | -0.06 (0.058) | -0.02 (0.498) | 0.02 (0.604) | -0.36 (<0.001) | -0.20 (<0.001) |  |  |  |
| Age in years | -0.06 (0.059) | 0.01 (0.780) | -0.03 (0.307) | -0.04 (0.263) | -0.01 (0.869) | -0.09 (0.004) | -0.01 (0.763) | 0.01 (0.685) | -0.02 (0.497) | 0.12 (0.001) | 0.05 (0.085) | 0.01 (0.825) | 0.08 (0.005) |  |  |
| Wealth quintile | 0.02 (0.485) | -0.04 (0.183) | 0.07 (0.036) | 0.03 (0.334) | 0.09 (0.005) | 0.02 (0.432) | -0.04 (0.208) | 0.09 (0.004) | -0.03 (0.319) | 0.01 (0.716) | 0.53 (<0.001) | 0.28 (<0.001) | -0.50 (<0.001) | -0.11 (<0.001) |  |
| Rural | -0.01 (0.645) | 0.05 (0.088) | 0.04 (0.197) | -0.08 (0.023) | 0.06 (0.051) | 0.05 (0.089) | -0.01 (0.784) | -0.03 (0.368) | -0.02 (0.463) | 0.01 (0.747) | -0.02 (0.536) | -0.01 (0.878) | -0.01 (0.795) | -0.09 (0.001) | -0.05 (0.113) |
|  |  |  |  |  |  |  |  |  |  |  |  |  |  |  |  |
| ^1^Food insecurity scale is out of 8 with increasing scores representing increasing food insecurity. Higher WASH and wealth quintiles represent cleaner and wealthier households, respectively. Ferritin was adjusted for CRP and AGP using BRINDA method. sTfR was adjusted for AGP using BRINDA method. Zinc was adjusted for CRP and AGP, time of processing, and fasting level. | | | | | | | | | | | | | | | |

| Supplemental Table 3: Standardized direct, indirect, and total effects for path model in preschool children living in Uttar Pradesh (N=507)^1^ | | | | | | | | |
| --- | --- | --- | --- | --- | --- | --- | --- | --- |
|  |  |  | **Standardized Coefficient (SE)** | | | | | |
| **Dependent variables** | **Predictors** |  | **Direct Effect** | **p-value** | **Indirect effect** | **p-value** | **Total effect** | **p-value** |
| Hemoglobin | Zinc |  | -0.054 | 0.136 | - | - | -0.054 | 0.136 |
|  | Folate |  | -0.169 | <0.001 | - | - | -0.169 | <0.001 |
|  | Vitamin B12 |  | -0.062 | 0.198 | - | - | -0.062 | 0.198 |
|  | Retinol |  | 0.067 | 0.034 | - | - | 0.067 | 0.034 |
|  | Ferritin |  | 0.492 | <0.001 | - | - | 0.492 | <0.001 |
|  | CRP |  | -0.004 | 0.945 | 0.111 | 0.001 | 0.107 | 0.086 |
|  | AGP |  | -0.200 | 0.002 | 0.040 | 0.216 | -0.159 | 0.033 |
|  | WASH |  | 0.063 | 0.162 | -0.019 | 0.549 | 0.044 | 0.443 |
|  | Dietary diversity |  | - | - | -0.060 | 0.052 | -0.060 | 0.052 |
|  | Food insecurity |  | - | - | 0.041 | 0.302 | 0.041 | 0.302 |
| Ferritin |  |  |  |  |  |  |  |  |
|  | Dietary diversity |  | -0.087 | 0.047 | - | - | -0.087 | 0.047 |
|  | Food insecurity |  | 0.031 | 0.682 | - | - | 0.031 | 0.682 |
|  | CRP |  | 0.212 | 0.002 | - | - | 0.212 | 0.002 |
|  | AGP |  | 0.139 | 0.016 | - | - | 0.139 | 0.016 |
|  | WASH |  | -0.011 | 0.837 | -0.062 | 0.055 | -0.073 | 0.181 |
| Retinol | Dietary diversity |  | 0.022 | 0.653 | - | - | 0.022 | 0.653 |
|  | Food insecurity |  | -0.111 | 0.014 | - | - | -0.111 | 0.014 |
|  | WASH |  | -0.009 | 0.857 | 0.060 | 0.006 | 0.051 | 0.316 |
|  | CRP |  | -0.074 | 0.328 | - | - | -0.074 | 0.328 |
|  | AGP |  | -0.098 | 0.119 | - | - | -0.098 | 0.119 |
| Vitamin B12 | Dietary diversity |  | 0.078 | 0.184 | - | - | 0.078 | 0.184 |
|  | Food insecurity |  | -0.166 | 0.008 | - | - | -0.166 | 0.008 |
|  | CRP |  | -0.012 | 0.036 | - | - | -0.012 | 0.036 |
|  | AGP |  | 0.015 | 0.013 | - | - | 0.015 | 0.013 |
|  | WASH |  | -0.033 | 0.709 | 0.059 | 0.010 | 0.026 | 0.773 |
| Folate | Dietary diversity |  | 0.070 | 0.189 | - | - | 0.070 | 0.189 |
|  | Food insecurity |  | -0.104 | 0.021 | - | - | -0.104 | 0.021 |
|  | CRP |  | -0.036 | 0.510 | - | - | -0.036 | 0.510 |
|  | AGP |  | 0.119 | 0.095 | - | - | 0.119 | 0.095 |
|  | WASH |  | 0.017 | 0.827 | 0.033 | 0.078 | 0.050 | 0.476 |
| Zinc | Dietary diversity |  | 0.042 | 0.457 | - | - | 0.042 | 0.457 |
|  | Food insecurity |  | -0.101 | 0.139 | - | - | -0.101 | 0.139 |
|  | WASH |  | 0.101 | 0.874 | 0.056 | 0.082 | 0.066 | 0.207 |
|  | CRP |  | 0.029 | 0.514 | - | - | 0.029 | 0.514 |
|  | AGP |  | -0.145 | 0.028 | - | - | -0.145 | 0.028 |
| AGP | WASH |  | -0.132 | 0.029 | - | - | -0.132 | 0.029 |
| CRP | WASH |  | -0.096 | 0.115 | - | - | -0.096 | 0.115 |
| Dietary diversity | WASH |  | 0.152 | 0.012 | - | - | 0.152 | 0.012 |
| Food insecurity | WASH |  | -0.332 | <0.001 | - | - | -0.332 | <0.001 |
| ^1^ Values are β coefficients (SE). All adjusted for clustering. Adjusted analyses accounting for age of child in months. Estimates of zero are not shown. | | | | | | | | |

| Supplemental Table 4: Unstandardized direct, indirect, and total effects for path model in preschool children living in Uttar Pradesh (N=507)^1^ | | | | | | | | |
| --- | --- | --- | --- | --- | --- | --- | --- | --- |
|  |  |  | **Unstandardized Coefficient (95% CI))** | | | | | |
| **Dependent variables** | **Predictors** |  | **Direct Effect** | **p-value** | **Indirect effect** | **p-value** | **Total effect** | **p-value** |
| Hemoglobin (g/dL) | ln Zinc (umol/L) |  | -0.190 (-0.439, 0.060) | 0.136 | - | - | -0.190 (-0.439, 0.596) | 0.136 |
|  | ln Folate (nmol/L) |  | -0.501 (-0.738, -0.264) | <0.001 | - | - | -0.501 (-0.738, -0.264) | <0.001 |
|  | ln Vitamin B12 (pmol/L) |  | -0.223 (-0.562, 0.116) | 0.198 | - | - | -0.223 (-0.562, 0.116) | 0.198 |
|  | ln Retinol (umol/L) |  | 0.158 (0.012, 0.304) | 0.034 | - | - | 0.158 (0.117, 0.304) | 0.034 |
|  | Ferritin (ug/L) |  | 0.901 (0.682, 1.119) | <0.001 | - | - | 0.901 (0.682, 1.119) | <0.001 |
|  | ln CRP (mg/L) |  | -0.033 (-0.096, 0.089) | 0.945 | 0.087 (0.035, 0.139) | 0.001 | 0.084 (-0.012, 0.179) | 0.086 |
|  | ln AGP (g/L) |  | -0.775 (-1.260, -0.290) | 0.002 | 0.157 (-0.092, 0.406) | 0.216 | -0.618 (-1.186, -0.050) | 0.033 |
|  | WASH |  | 0.072 (-0.029, 0.172) | 0.162 | -0.022 (-0.094, 0.050) | 0.549 | 0.050 (-0.077, 0.177) | 0.443 |
|  | Dietary diversity |  | - | - | -0.077 (-0.154, 0.001) | 0.052 | -0.077 (-0.154, 0.001) | 0.052 |
|  | Food insecurity |  | - | - | 0.027 (-0.025, 0.080) | 0.302 | 0.028 (-0.024, 0.079) | 0.298 |
| Ferritin (ug/L) | Dietary diversity |  | -0.060 (-0.120, -0.001) | 0.047 | - | - | -0.060 (-0.120, -0.001) | 0.047 |
|  | Food insecurity |  | 0.011 (-0.043, 0.066) | 0.682 | - | - | 0.011 (-0.043, 0.066) | 0.682 |
|  | ln CRP (mg/L) |  | 0.090 (0.034, 0.146) | 0.002 | - | - | 0.090 (0.034, 0.146) | 0.002 |
|  | ln AGP (g/L) |  | 0.294 (0.054, 0.535) | 0.016 | - | - | 0.294 (0.054, 0.535) | 0.016 |
|  | WASH |  | -0.007 (-0.070, 0.057) | 0.837 | -0.039 (-0.078, 0.001) | 0.055 | -0.045 (-0.111, 0.021) | 0.181 |
| ln Retinol (umol/L) | Dietary diversity |  | 0.012 (-0.039, 0.063) | 0.653 | - | - | 0.012 (-0.039, 0.063) | 0.653 |
|  | Food insecurity |  | -0.031 (-0.056, -0.006) | 0.014 | - | - | -0.031 (-0.056, -0.006) | 0.014 |
|  | ln CRP (mg/L) |  | -0.024 (-0.073, 0.024) | 0.328 | - | - | -0.024 (-0.073, 0.024) | 0.328 |
|  | ln AGP (g/L) |  | -0.161 (-0.363, 0.041) | 0.119 | - | - | -0.161 (-0.363, 0.041) | 0.119 |
|  | WASH |  | -0.004 (-0.053, 0.044) | 0.857 | 0.029 (0.008, 0.049) | 0.006 | 0.024 (-0.023, 0.072) | 0.316 |
| ln Vitamin B12 (pmol/L) | Dietary diversity |  | -0.031 (-0.010, 0.071) | 0.137 | - | - | 0.031 (-0.010, 0.071) | 0.137 |
|  | Food insecurity |  | -0.031 (-0.053, -0.009) | 0.005 | - | - | -0.031 (-0.053, -0.009) | 0.005 |
|  | ln CRP (mg/L) |  | -0.026 (-0.051, -0.002) | 0.036 | - | - | -0.026 (-0.051, -0.002) | 0.036 |
|  | ln AGP (g/L) |  | 0.158 (0.034, 0.282) | 0.013 | - | - | 0.158 (0.034, 0.282) | 0.013 |
|  | WASH |  | -0.010 (-0.065, 0.044) | 0.709 | 0.019 (0.004, 0.033) | 0.01 | 0.008 (-0.047, 0.063) | 0.773 |
| ln Folate (nmol/L) | Dietary diversity |  | 0.030 (-0.015, 0.076) | 0.189 | - | - | 0.030 (-0.015, 0.076) | 0.189 |
|  | Food insecurity |  | -0.023 (-0.043, -0.003) | 0.022 | - | - | -0.023 (-0.043, -0.003) | 0.022 |
|  | ln CRP (mg/L) |  | -0.010 (-0.038, 0.019) | 0.510 | - | - | -0.010 (-0.038, 0.019) | 0.510 |
|  | ln AGP (g/L) |  | 0.156 (-0.027, 0.339) | 0.095 | - | - | 0.156 (-0.027, 0.339) | 0.095 |
|  | WASH |  | 0.002 (-0.052, 0.055) | 0.947 | 0.013 (-0.001, 0.026) | 0.078 | 0.019 (-0.033, 0.710) | 0.476 |
| ln Zinc (umol/L) | Dietary diversity |  | 0.015 (-0.024, 0.054) | 0.457 | - | - | 0.015 (-0.024, 0.054) | 0.457 |
|  | Food insecurity |  | -0.019 (-0.045, 0.006) | 0.139 | - | - | -0.019 (-0.045, 0.006) | 0.139 |
|  | ln CRP (mg/L) |  | 0.006 (-0.013, 0.025) | 0.514 | - | - | 0.006 (-0.013, 0.025) | 0.514 |
|  | ln AGP (g/L) |  | -0.160 (-0.302, -0.017) | 0.028 | - | - | -0.16 (-0.302, -0.017) | 0.028 |
|  | WASH |  | 0.003 (-0.037, 0.043) | 0.874 | 0.018 (-0.002, 0.038) | 0.082 | 0.021 (-0.012, 0.054) | 0.207 |
| ln AGP (g/L) | WASH |  | -0.039 (-0.073, -0.004) | 0.029 | - | - | -0.039 (-0.073, -0.004) | 0.029 |
| ln CRP (mg/L) | WASH |  | -0.140 (-0.315, 0.034) | 0.115 | - | - | -0.140 (-0.315, 0.034) | 0.115 |
| Dietary diversity | WASH |  | 0.135 (0.030, 0.239) | 0.012 | - | - | 0.135 (0.030, 0.239) | 0.012 |
| Food insecurity | WASH |  | -0.563 (-0.687, -0.438) | <0.001 | - | - | -0.563 (-0.687, -0.438) | <0.001 |
| ^1^ Values are β coefficients (95% CI). All adjusted for clustering. Adjusted analyses accounting for age of child in months. Estimates of zero are not shown. | | | | | | | | |

| Supplemental Table 5: Standardized direct, indirect, and total effects for path model in mothers of reproductive age living in Uttar Pradesh (N=798)^1^ | | | | | | | | | | |
| --- | --- | --- | --- | --- | --- | --- | --- | --- | --- | --- |
|  |  |  | **Standardized Coefficient** | | | | | | | |
| **Dependent variables** | **Predictors** |  | **Direct Effect** | **p-value** | **Indirect effect** | **p-value** | **Total effect** | **p-value** |  |  |
| Hemoglobin | Zinc |  | 0.054 | 0.204 | - | - | 0.054 | 0.204 |  |  |
|  | Folate |  | -0.103 | 0.076 | - | - | -0.010 | 0.076 |  |  |
|  | Vitamin B12 |  | 0.706 | 0.053 | - | - | 0.706 | 0.053 |  |  |
|  | Retinol |  | 0.055 | 0.080 | - | - | 0.055 | 0.080 |  |  |
|  | Ferritin |  | 0.537 | <0.001 | - | - | 0.537 | <0.001 |  |  |
|  | CRP |  | -0.053 | 0.201 | 0.072 | 0.006 | 0.019 | 0.696 |  |  |
|  | AGP |  | -0.006 | 0.915 | 0.060 | 0.214 | 0.054 | 0.504 |  |  |
|  | WASH |  | - | - | 0.027 | 0.308 | 0.027 | 0.308 |  |  |
|  | Dietary diversity |  | - | - | -0.011 | 0.791 | -0.011 | 0.791 |  |  |
|  | Food insecurity |  | - | - | -0.027 | 0.426 | -0.027 | 0.426 |  |  |
|  |  |  |  |  |  |  |  |  |  |  |
| Ferritin | Dietary diversity |  | 0.005 | 0.945 | - | - | 0.005 | 0.945 |  |  |
|  | Food insecurity |  | -0.048 | 0.447 | - | - | -0.048 | 0.447 |  |  |
|  | CRP |  | 0.142 | 0.006 | - | - | 0.142 | 0.006 |  |  |
|  | AGP |  | 0.104 | 0.173 | - | - | 0.104 | 0.173 |  |  |
|  | WASH |  | 0.022 | 0.664 | 0.047 | 0.126 | 0.070 | 0.100 |  |  |
| Retinol | Dietary diversity |  | 0.077 | 0.042 | - | - | 0.076 | 0.042 |  |  |
|  | Food insecurity |  | -0.035 | 0.461 | - | - | -0.035 | 0.461 |  |  |
|  | WASH |  | -0.008 | 0.836 | 0.036 | 0.111 | 0.029 | 0.470 |  |  |
|  | CRP (mg/L) |  | 0.078 | 0.168 | - | - | 0.078 | 0.168 |  |  |
|  | AGP (g/L) |  | -0.059 | 0.344 | - | - | -0.059 | 0.344 |  |  |
| Vitamin B12 | Dietary diversity |  | 0.04 | 0.383 | - | - | 0.043 | 0.358 |  |  |
|  | Food insecurity |  | 0.018 | 0.667 | - | - | 0.016 | 0.697 |  |  |
|  | CRP (mg/L) |  | -0.015 | 0.783 | - | - | -0.015 | 0.783 |  |  |
|  | AGP (g/L) |  | 0.111 | 0.021 | - | - | 0.111 | 0.021 |  |  |
|  | WASH |  | 0.057 | 0.105 | 0.005 | 0.803 | 0.062 | 0.154 |  |  |
| Folate | Dietary diversity |  | 0.156 | 0.002 | - | - | 0.156 | 0.002 |  |  |
|  | Food insecurity |  | -0.032 | 0.421 | - | - | -0.032 | 0.421 |  |  |
|  | CRP (mg/L) |  | 0.094 | 0.004 | - | - | 0.094 | 0.004 |  |  |
|  | AGP (g/L) |  | -0.015 | 0.757 | - | - | -0.015 | 0.757 |  |  |
|  | WASH |  | 0.019 | 0.736 | 0.056 | 0.005 | 0.742 | 0.207 |  |  |
| Zinc | Dietary diversity |  | -0.078 | 0.076 | - | - | -0.078 | 0.076 |  |  |
|  | Food insecurity |  | -0.076 | 0.216 | - | - | -0.076 | 0.216 |  |  |
|  | WASH |  | -0.019 | 0.637 | 0.020 | 0.45 | 0.000 | 0.995 |  |  |
|  | CRP |  | 0.053 | 0.192 | - | - | 0.053 | 0.192 |  |  |
|  | AGP |  | -0.038 | 0.505 | - | - | -0.038 | 0.505 |  |  |
| AGP | WASH |  | 0.059 | 0.106 | - | - | 0.059 | 0.106 |  |  |
| CRP | WASH |  | 0.165 | 0.010 | - | - | 0.165 | 0.010 |  |  |
| Dietary diversity | WASH |  | 0.187 | <0.001 | - | - | 0.187 | <0.001 |  |  |
| Food insecurity | WASH |  | -0.362 | <0.001 | - | - | -0.362 | <0.001 |  |  |
| ^1^ Values are β coefficients (SE). All adjusted for clustering. Adjusted analyses accounting for age of child in months. Estimates of zero are not shown. | | | | | | | | | | |

| Supplemental Table 6: Unstandardized direct, indirect, and total effects for path model in mothers of reproductive age living in Uttar Pradesh (N=798)^1^ | | | | | | | |  |
| --- | --- | --- | --- | --- | --- | --- | --- | --- |
|  |  |  | **Unstandardized Coefficient (95% CI)** | | | | |  |
| **Dependent variables** | **Predictors** |  | **Direct Effect** | **p-value** | **Indirect effect** | **p-value** | **Total effect** | **p-value** |
| Hemoglobin (g/dL) | ln Zinc (umol/L) |  | 0.228 (-0.124, 0.581) | 0.204 | - | - | 0.228 (-0.124, 0.581) | 0.204 |
|  | ln Folate (nmol/L) |  | -0.270 (-0.567, 0.028) | 0.076 | - | - | -0.270 (-0.567, 0.028) | 0.076 |
|  | ln Vitamin B12 (pmol/L) |  | 0.204 (-0.003, 0.411) | 0.053 | - | - | 0.204 (-0.003, 0.411) | 0.053 |
|  | ln Retinol (umol/L) |  | 0.155 (-0.185, 0.328) | 0.080 | - | - | 0.155 (-0.185, 0.328) | 0.080 |
|  | Ferritin (ug/L) |  | 1.033 (0.800, 1.265) | <0.001 | - | - | 1.033 (0.800, 1.265) | <0.001 |
|  | ln CRP (mg/L) |  | -0.052 (-0.131, 0.028) | 0.201 | 0.706 (0.021, 0.121) | 0.006 | 0.019 (-0.075, 0.113) | 0.696 |
|  | ln AGP (g/L) |  | -0.029 (-0.564, 0.505) | 0.915 | 0.289 (-0.167, 0.745) | 0.214 | 0.260 (-0.503, 1.023) | 0.504 |
|  | WASH |  | - | - | 0.032 (-0.030, 0.094) | 0.308 | 0.032 (-0.030, 0.094) | 0.308 |
|  | Dietary diversity |  | - | - | -0.017 (-0.139, 0.106) | 0.791 | -0.016 (-0.139, 0.107) | 0.795 |
|  | Food insecurity |  | - | - | -0.018 (-0.063, 0.026) | 0.426 | -0.018 (-0.063, 0.027) | 0.426 |
| Ferritin (ug/L) | Dietary diversity |  | 0.004 (-0.101, 0.109) | 0.945 | - | - | 0.004 (-0.101, 0.109) | 0.945 |
|  | Food insecurity |  | -0.017 (-0.060, 0.026) | 0.447 | - | - | -0.017 (-0.060, 0.026) | 0.447 |
|  | ln CRP (mg/L) |  | 0.718 (0.020, 0.123) | 0.006 | - | - | 0.718 (0.020, 0.123) | 0.006 |
|  | ln AGP (g/L) |  | 0.261 (-0.114, 0.636) | 0.173 | - | - | 0.261 (-0.114, 0.636) | 0.173 |
|  | WASH |  | 0.014 (-0.049, 0.076) | 0.664 | 0.030 (-0.008, 0.068) | 0.126 | 0.044 (-0.008, 0.096) | 0.100 |
| ln Retinol (umol/L) | Dietary diversity |  | 0.041 (0.002, 0.082) | 0.042 | - | - | 0.041 (0.002, 0.080) | 0.038 |
|  | Food insecurity |  | -0.008 (-0.030, 0.014) | 0.461 | - | - | -0.008 (-0.030, 0.014) | 0.454 |
|  | WASH |  | -0.003 (-0.034, 0.028) | 0.836 | 0.016 (-0.004, 0.035) | 0.111 | 0.012 (-0.021, 0.046) | 0.470 |
|  | CRP (mg/L) |  | 0.027 (-0.011, 0.065) | 0.168 | - | - | 0.027 (-0.011, 0.065) | 0.168 |
|  | AGP (g/L) |  | -0.101 (-0.311, 0.108) | 0.344 | - | - | -0.101 (-0.311, 0.108) | 0.344 |
| ln Vitamin B12 (pmol/L) | Dietary diversity |  | 0.021 (-0.026, 0.068) | 0.383 | - | - | 0.021 (-0.026, 0.071) | 0.383 |
|  | Food insecurity |  | 0.004 (-0.015, 0.023) | 0.667 | - | - | 0.004 (-0.015, 0.023) | 0.697 |
|  | CRP (mg/L) |  | -0.005 (-0.041, 0.031) | 0.783 | - | - | -0.005 (-0.041, 0.031) | 0.783 |
|  | AGP (g/L) |  | 0.186 (0.028, 0.345) | 0.021 | - | - | 0.186 (0.028, 0.345) | 0.021 |
|  | WASH |  | 0.024 (-0.005, 0.053) | 0.105 | 0.002 (-0.015, 0.019) | 0.803 | 0.026 (-0.010, 0.062) | 0.154 |
| ln Folate (nmol/L) | Dietary diversity |  | 0.092 (0.033, 0.150) | 0.002 | - | - | 0.092 (0.034, 0.150) | 0.002 |
|  | Food insecurity |  | -0.008 (-0.029, 0.012) | 0.421 | - | - | -0.008 (-0.029, 0.012) | 0.421 |
|  | CRP (mg/L) |  | 0.035 (0.011, 0.059) | 0.004 | - | - | 0.035 (0.011, 0.059) | 0.004 |
|  | AGP (g/L) |  | -0.027 (-0.199, 0.145) | 0.757 | - | - | -0.027 (-0.199, 0.145) | 0.757 |
|  | WASH |  | 0.009 (-0.042, 0.059) | 0.736 | 0.026 (0.008, 0.044) | 0.005 | 0.034 (-0.019, 0.088) | 0.207 |
| ln Zinc (umol/L) | Dietary diversity |  | -0.028 (-0.059, 0.003) | 0.076 | - | - | -0.028 (-0.059, 0.003) | 0.076 |
|  | Food insecurity |  | -0.012 (-0.031, 0.007) | 0.216 | - | - | -0.012 (-0.031, 0.007) | 0.216 |
|  | ln CRP (mg/L) |  | 0.012 (-0.006, 0.030) | 0.192 | - | - | 0.012 (-0.006, 0.030) | 0.192 |
|  | ln AGP (g/L) |  | -0.043 (-0.171, 0.084) | 0.505 | - | - | -0.043 (-0.171, 0.084) | 0.505 |
|  | WASH |  | -0.005 (-0.028, 0.017) | 0.637 | 0.006 (-0.009, 0.020) | 0.450 | 0.000 (-0.018, 0.018) | 0.995 |
| ln AGP (g/L) | WASH |  | 0.015 (-0.003, 0.033) | 0.106 | - | - | 0.015 (-0.003, 0.033) | 0.106 |
| ln CRP (mg/L) | WASH |  | 0.204 (0.050, 0.359) | 0.010 | - | - | 0.204 (0.050, 0.359) | 0.010 |
| Dietary diversity | WASH |  | 0.148 (0.079, 0.217) | <0.001 | - | - | 0.148 (0.079, 0.217) | <0.001 |
| Food insecurity | WASH |  | -0.650 (-0.746, -0.554) | <0.001 | - | - | -0.650 (-0.746, -0.555) | <0.001 |
| ^1^ Values are β coefficients (95% CI). All adjusted for clustering. Adjusted analyses accounting for age of child in months. Estimates of zero are not shown. * p<0.05. ** p<0.01. *** p<0.001 | | | | | | | |  |

| Supplemental Table 7: Standardized direct, indirect, and total effects for path model, among mothers with β-thalassemia measurement (N=455)^1^ | | | | | | | | | | |
| --- | --- | --- | --- | --- | --- | --- | --- | --- | --- | --- |
|  |  |  | **Standardized Coefficient** | | | | | | | |
| **Dependent variables** | **Predictors** |  | **Direct Effect** | **p-value** | **Indirect effect** | **p-value** | **Total effect** | **p-value** |  |  |
| Hemoglobin | Zinc |  | -0.015 | 0.747 | - | - | -0.015 | 0.747 |  |  |
|  | Folate |  | -0.083 | 0.298 | - | - | -0.083 | 0.298 |  |  |
|  | Vitamin B12 |  | 0.070 | 0.129 | - | - | 0.070 | 0.129 |  |  |
|  | Retinol |  | 0.069 | 0.108 | - | - | 0.069 | 0.108 |  |  |
|  | Ferritin |  | 0.582 | <0.001 | - | - | 0.582 | <0.001 |  |  |
|  | CRP |  | -0.031 | 0.561 | 0.081 | 0.021 | 0.049 | 0.382 |  |  |
|  | AGP |  | 0.021 | 0.734 | 0.006 | 0.912 | 0.027 | 0.738 |  |  |
|  | WASH |  | - | - | 0.027 | 0.168 | 0.027 | 0.168 |  |  |
|  | Dietary diversity |  | - | - | -0.034 | 0.290 | -0.034 | 0.290 |  |  |
|  | β Thalassemia |  | -0.038 | 0.019 | 0.006 | 0.726 | -0.031 | 0.162 |  |  |
|  | Food insecurity |  | - | - | -0.016 | 0.812 | -0.016 | 0.812 |  |  |
|  |  |  |  |  |  |  |  |  |  |  |
| Ferritin | Dietary diversity |  | -0.037 | 0.477 | - | - | -0.037 | 0.477 |  |  |
|  | Food insecurity |  | -0.028 | 0.799 | - | - | -0.028 | 0.799 |  |  |
|  | CRP |  | 0.145 | 0.032 | - | - | 0.145 | 0.032 |  |  |
|  | AGP |  | 0.006 | 0.953 | - | - | 0.006 | 0.953 |  |  |
|  | β Thalassemia |  | 0.011 | 0.718 | - | - | 0.011 | 0.718 |  |  |
|  | WASH |  | 0.027 | 0.651 | 0.031 | 0.548 | 0.058 | 0.035 |  |  |
| Retinol | Dietary diversity |  | 0.040 | 0.438 | - | - | 0.040 | 0.438 |  |  |
|  | Food insecurity |  | -0.012 | 0.845 | - | - | -0.012 | 0.845 |  |  |
|  | WASH |  | -0.003 | 0.956 | 0.022 | 0.544 | 0.018 | 0.787 |  |  |
|  | CRP (mg/L) |  | 0.074 | 0.303 | - | - | 0.074 | 0.303 |  |  |
|  | AGP (g/L) |  | -0.013 | 0.843 | - | - | -0.013 | 0.843 |  |  |
| Vitamin B12 | Dietary diversity |  | -0.032 | 0.535 | - | - | -0.032 | 0.535 |  |  |
|  | Food insecurity |  | 0.033 | 0.442 | - | - | 0.033 | 0.442 |  |  |
|  | CRP (mg/L) |  | -0.007 | 0.909 | - | - | -0.007 | 0.909 |  |  |
|  | AGP (g/L) |  | 0.085 | 0.274 | - | - | 0.085 | 0.274 |  |  |
|  | WASH |  | 0.048 | 0.354 | -0.013 | 0.546 | 0.034 | 0.424 |  |  |
| Folate | Dietary diversity |  | 0.175 | 0.003 | - | - | 0.175 | 0.003 |  |  |
|  | Food insecurity |  | 0.009 | 0.875 | - | - | 0.009 | 0.875 |  |  |
|  | CRP (mg/L) |  | 0.094 | 0.001 | - | - | 0.094 | 0.001 |  |  |
|  | AGP (g/L) |  | 0.018 | 0.808 | - | - | 0.018 | 0.808 |  |  |
|  | WASH |  | 0.052 | 0.551 | 0.036 | 0.185 | 0.088 | 0.275 |  |  |
| Zinc | Dietary diversity |  | -0.119 | 0.054 | - | - | -0.119 | 0.054 |  |  |
|  | Food insecurity |  | 0.036 | 0.586 | - | - | 0.036 | 0.586 |  |  |
|  | WASH |  | 0.030 | 0.572 | -0.024 | 0.372 | 0.006 | 0.894 |  |  |
|  | CRP |  | 0.033 | 0.591 | - | - | 0.033 | 0.591 |  |  |
|  | AGP |  | 0.023 | 0.739 | - | - | 0.023 | 0.739 |  |  |
| AGP | WASH |  | 0.076 | 0.222 | - | - | 0.076 | 0.222 |  |  |
| CRP | WASH |  | 0.164 | 0.076 | - | - | 0.164 | 0.076 |  |  |
| Dietary diversity | WASH |  | 0.131 | 0.001 | - | - | 0.131 | 0.001 |  |  |
| Food insecurity | WASH |  | -0.434 | <0.001 | - | - | -0.434 | <0.001 |  |  |
| ^1^ Values are β coefficients (SE). All adjusted for clustering. Adjusted analyses accounting for age of child in months. Estimates of zero are not shown. | | | | | | | | | | |
| Model fit statistics: CFI=0.918, TLI=0.795, RMSEA=0.053, SRMR=0.041 | | | | | | | |  |  |  |

| Supplemental Table 8: Unstandardized direct, indirect, and total effects for path model among mothers with β-thalassemia measurement (N=455)^1^ | | | | | | | |  |
| --- | --- | --- | --- | --- | --- | --- | --- | --- |
|  |  |  | **Unstandardized Coefficient (95% CI)** | | | | |  |
| **Dependent variables** | **Predictors** |  | **Direct Effect** | **p-value** | **Indirect effect** | **p-value** | **Total effect** | **p-value** |
| Hemoglobin (g/dL) | ln Zinc (umol/L) |  | -0.076 (-0.539, 0.387) | 0.747 | - | - | -0.076 (-0.539, 0.387) | 0.747 |
|  | ln Folate (nmol/L) |  | -0.218 (-0.629, 0.193) | 0.298 | - | - | -0.218 (-0.629, 0.193) | 0.298 |
|  | ln Vitamin B12 (pmol/L) |  | 0.208 (-0.061, 0.476) | 0.129 | - | - | 0.208 (-0.061, 0.476) | 0.129 |
|  | ln Retinol (umol/L) |  | 0.203 (-0.045, 0.450) | 0.108 | - | - | 0.203 (-0.045, 0.450) | 0.108 |
|  | Ferritin (ug/L) |  | 1.206 (0.951, 1.461) | <0.001 | - | - | 1.206 (0.951, 1.461) | <0.001 |
|  | ln CRP (mg/L) |  | -0.034 (-0.149, 0.081) | 0.561 | 0.088 (0.014, 0.163) | 0.021 | 0.054 (-0.067, 0.176) | 0.382 |
|  | ln AGP (g/L) |  | 0.107 (-0.051, 0.728) | 0.734 | 0.033 (-0.559, 0.625) | 0.912 | 0.141 (-0.683, 0.964) | 0.738 |
|  | β Thalassemia |  | -0.522 (-0.959, -0.085) | 0.019 | 0.086 (-0.395, 0.568) | 0.726 | -0.436 (-1.048, 0.175) | 0.162 |
|  | WASH |  | - | - | 0.034 (-0.014, 0.083) | 0.168 | 0.034 (-0.014, 0.083) | 0.168 |
|  | Dietary diversity |  | - | - | -0.057 (-0.162, 0.048) | 0.29 | -0.057 (-0.162, 0.048) | 0.29 |
|  | Food insecurity |  | - | - | -0.011 (-0.106, 0.083) | 0.812 | -0.011 (-0.106, 0.083) | 0.812 |
| Ferritin (ug/L) | Dietary diversity |  | -0.030 (-0.113, 0.053) | 0.477 | - | - | -0.030 (-0.113, 0.053) | 0.477 |
|  | Food insecurity |  | -0.010 (-0.084, 0.064) | 0.799 | - | - | -0.010 (-0.084, 0.064) | 0.799 |
|  | ln CRP (mg/L) |  | 0.077 (0.007, 0.146) | 0.032 | - | - | 0.077 (0.007, 0.146) | 0.032 |
|  | ln AGP (g/L) |  | 0.139 (-0.451, 0.479) | 0.953 | - | - | 0.139 (-0.451, 0.479) | 0.953 |
|  | β Thalassemia |  | 0.071 (-0.316, 0.459) | 0.718 | - | - | 0.071 (-0.316, 0.459) | 0.718 |
|  | WASH |  | 0.017 (-0.055, 0.089) | 0.651 | 0.019 (-0.044, 0.083) | 0.548 | 0.036 (0.003, 0.069) | 0.035 |
| ln Retinol (umol/L) | Dietary diversity |  | 0.023 (-0.035, 0.081) | 0.438 | - | - | 0.023 (-0.035, 0.081) | 0.438 |
|  | Food insecurity |  | -0.003 (-0.032, 0.026) | 0.845 | - | - | -0.003 (-0.032, 0.026) | 0.845 |
|  | WASH |  | -0.001 (-0.054, 0.051) | 0.956 | 0.009 (-0.021, 0.040) | 0.111 | 0.008 (-0.050, 0.065) | 0.787 |
|  | CRP (mg/L) |  | 0.028 (-0.025, 0.080) | 0.303 | - | - | 0.028 (-0.025, 0.080) | 0.303 |
|  | AGP (g/L) |  | -0.023 (-0.249, 0.204) | 0.843 | - | - | -0.023 (-0.249, 0.204) | 0.843 |
| ln Vitamin B12 (pmol/L) | Dietary diversity |  | -0.018 (-0.075, 0.039) | 0.535 | - | - | -0.018 (-0.075, 0.039) | 0.535 |
|  | Food insecurity |  | 0.008 (-0.012, 0.028) | 0.442 | - | - | 0.008 (-0.012, 0.028) | 0.442 |
|  | CRP (mg/L) |  | -0.003 (-0.050, 0.044) | 0.909 | - | - | -0.003 (-0.050, 0.044) | 0.909 |
|  | AGP (g/L) |  | 0.149 (-0.117, 0.414) | 0.274 | - | - | 0.149 (-0.117, 0.414) | 0.274 |
|  | WASH |  | 0.021 (-0.023, 0.064) | 0.354 | -0.006 (-0.025, 0.013) | 0.546 | 0.015 (-0.021, 0.051) | 0.424 |
| ln Folate (nmol/L) | Dietary diversity |  | 0.111 (0.037, 0.185) | 0.003 | - | - | 0.111 (0.037, 0.185) | 0.003 |
|  | Food insecurity |  | 0.002 (-0.027, 0.031) | 0.875 | - | - | 0.002 (-0.027, 0.031) | 0.875 |
|  | CRP (mg/L) |  | 0.039 (0.015, 0.063) | 0.001 | - | - | 0.039 (0.015, 0.063) | 0.001 |
|  | AGP (g/L) |  | 0.036 (-0.256, 0.329) | 0.808 | - | - | 0.036 (-0.256, 0.329) | 0.808 |
|  | WASH |  | 0.025 (-0.058, 0.108) | 0.551 | 0.018 (-0.008, 0.044) | 0.185 | 0.043 (-0.034, 0.120) | 0.275 |
| ln Zinc (umol/L) | Dietary diversity |  | -0.040 (-0.080, 0.001) | 0.054 | - | - | -0.040 (-0.080, 0.001) | 0.054 |
|  | Food insecurity |  | 0.005 (-0.013, 0.023) | 0.586 | - | - | 0.005 (-0.013, 0.023) | 0.586 |
|  | ln CRP (mg/L) |  | 0.007 (-0.019, 0.033) | 0.591 | - | - | 0.007 (-0.019, 0.033) | 0.591 |
|  | ln AGP (g/L) |  | 0.024 (-0.118, 0.166) | 0.739 | - | - | 0.024 (-0.118, 0.166) | 0.739 |
|  | WASH |  | 0.008 (-0.019, 0.034) | 0.572 | -0.006 (-0.020, 0.007) | 0.372 | 0.002 (-0.022, 0.025) | 0.894 |
| ln AGP (g/L) | WASH |  | -0.779 (-0.911, -0.647) | <0.001 | - | - | -0.779 (-0.911, -0.647) | <0.001 |
| ln CRP (mg/L) | WASH |  | 0.191 (-0.020, 0.403) | 0.076 | - | - | 0.191 (-0.020, 0.403) | 0.076 |
| Dietary diversity | WASH |  | 0.101 (0.042, 0.160) | 0.001 | - | - | 0.101 (0.042, 0.160) | 0.001 |
| Food insecurity | WASH |  | -0.779 (-0.911, -0.647) | <0.001 | - | - | -0.779 (-0.911, -0.647) | <0.001 |
| ^1^ Values are β coefficients (95% CI). All adjusted for clustering. Adjusted analyses accounting for age of child in months. Estimates of zero are not shown. | | | | | | | |  |
| Model fit statistics: CFI=0.918, TLI=0.795, RMSEA=0.053, SRMR=0.041 | | | | | | | |  |

| Supplemental Table 9: Standardized direct, indirect, and total effects for path model in preschool children living in Uttar Pradesh using full information maximum likelihood (N=1238)^1^ | | | | | | | | |
| --- | --- | --- | --- | --- | --- | --- | --- | --- |
|  |  |  | **Standardized Coefficient (SE)** | | | | | |
| **Dependent variables** | **Predictors** |  | **Direct Effect** | **p-value** | **Indirect effect** | **p-value** | **Total effect** | **p-value** |
| Hemoglobin | Zinc |  | -0.014 | 0.730 | - | - | -0.014 | 0.730 |
|  | Folate |  | -0.142 | <0.001 | - | - | -0.142 | <0.001 |
|  | Vitamin B12 |  | -0.053 | 0.299 | - | - | -0.053 | 0.299 |
|  | Retinol |  | 0.073 | 0.001 | - | - | 0.073 | 0.001 |
|  | Ferritin |  | 0.491 | <0.001 | - | - | 0.491 | <0.001 |
|  | CRP |  | -0.018 | 0.721 | 0.099 | 0.008 | 0.081 | 0.135 |
|  | AGP |  | -0.179 | <0.001 | 0.064 | 0.046 | -0.114 | 0.037 |
|  | WASH |  | 0.028 | 0.433 | 0.002 | 0.919 | 0.304 | 0.382 |
|  | Dietary diversity |  | - | - | -0.017 | 0.505 | -0.017 | 0.505 |
|  | Household hunger |  | - | - | 0.000 | 0.996 | 0.000 | 0.996 |
| Ferritin |  |  |  |  |  |  |  |  |
|  | Dietary diversity |  | -0.029 | 0.506 | - | - | -0.029 | 0.506 |
|  | Household hunger |  | -0.033 | 0.561 | - | - | -0.033 | 0.561 |
|  | CRP |  | 0.203 | 0.001 | - | - | 0.203 | 0.001 |
|  | AGP |  | 0.175 | 0.005 | - | - | 0.175 | 0.005 |
|  | WASH |  | -0.006 | 0.895 | -0.025 | 0.29 | -0.030 | 0.492 |
| Retinol | Dietary diversity |  | 0.105 | 0.040 | - | - | 0.105 | 0.040 |
|  | Household hunger |  | -0.023 | 0.626 | - | - | -0.023 | 0.626 |
|  | WASH |  | <0.001 | 0.995 | 0.034 | 0.078 | 0.034 | 0.506 |
|  | CRP |  | -0.083 | 0.191 | - | - | -0.083 | 0.191 |
|  | AGP |  | -0.066 | 0.297 | - | - | -0.066 | 0.297 |
| Vitamin B12 | Dietary diversity |  | 0.066 | 0.337 | - | - | 0.066 | 0.337 |
|  | Household hunger |  | -0.115 | 0.017 | - | - | -0.115 | 0.017 |
|  | CRP |  | -0.118 | 0.015 | - | - | -0.118 | 0.015 |
|  | AGP |  | -0.179 | <0.001 | - | - | -0.179 | <0.001 |
|  | WASH |  | -0.006 | 0.940 | 0.036 | 0.060 | 0.030 | 0.668 |
| Folate | Dietary diversity |  | 0.044 | 0.169 | - | - | 0.044 | 0.169 |
|  | Household hunger |  | -0.079 | 0.105 | - | - | -0.079 | 0.105 |
|  | CRP |  | 0.004 | 0.933 | - | - | 0.004 | 0.933 |
|  | AGP |  | 0.058 | 0.288 | - | - | 0.058 | 0.288 |
|  | WASH |  | -0.005 | 0.942 | 0.027 | 0.066 | 0.023 | 0.69 |
| Zinc | Dietary diversity |  | 0.061 | 0.179 | - | - | 0.061 | 0.179 |
|  | Household hunger |  | -0.065 | 0.128 | - | - | -0.065 | 0.128 |
|  | WASH |  | 0.017 | 0.709 | 0.046 | 0.042 | 0.063 | 0.166 |
|  | CRP |  | 0.015 | 0.717 | - | - | 0.015 | 0.717 |
|  | AGP |  | -0.154 | 0.010 | - | - | -0.154 | 0.01 |
| AGP | WASH |  | -0.107 | 0.019 | - | - | -0.107 | 0.019 |
| CRP | WASH |  | -0.071 | 0.172 | - | - | -0.071 | 0.172 |
| Dietary diversity | WASH |  | 0.120 | 0.005 | - | - | 0.120 | 0.005 |
| Household hunger | WASH |  | -0.359 | <0.001 | - | - | -0.359 | <0.001 |
| 1 Values are β coefficients (SE). All adjusted for clustering. Adjusted analyses accounting for age of child in months. Estimates of zero are not shown. * p<0.05. ** p<0.01. *** p<0.001 | | | | | | | | |
| Fit statistics: CFI=0.938, TLI=0.742, RMSEA=0.064, SRMR=0.031 | | | | | | | | |

| Supplemental Table 10: Unstandardized direct, indirect, and total effects for path model in preschool children living in Uttar Pradesh, using full information maximum likelihood (N=1238)^1^ | | | | | | | | |
| --- | --- | --- | --- | --- | --- | --- | --- | --- |
|  |  |  | **Unstandardized Coefficient (95% CI))** | | | | | |
| **Dependent variables** | **Predictors** |  | **Direct Effect** | **p-value** | **Indirect effect** | **p-value** | **Total effect** | **p-value** |
| Hemoglobin (g/dL) | ln Zinc (umol/L) |  | -0.051 (-0.344, 0.241) | 0.730 | - | - | -0.051 (-0.344, 0.241) | 0.730 |
|  | ln Folate (nmol/L) |  | -0.393 (-0.573, -0.214) | <0.001 | - | - | -0.393 (-0.573, -0.214) | <0.001 |
|  | ln Vitamin B12 (pmol/L) |  | -0.181 (-0.522, 0.160) | 0.299 | - | - | -0.181 (-0.522, 0.160) | 0.299 |
|  | ln Retinol (umol/L) |  | 0.170 (0.072, 0.268) | 0.001 | - | - | 0.170 (0.072, 0.268) | 0.001 |
|  | Ferritin (ug/L) |  | 0.868 (0.646, 1.089) | <0.001 | - | - | 0.868 (0.646, 1.089) | <0.001 |
|  | ln CRP (mg/L) |  | -0.015 (-0.097, 0.067) | 0.721 | 0.082 (0.022, 0.141) | 0.008 | 0.067 (-0.021, 0.154) | 0.135 |
|  | ln AGP (g/L) |  | -0.674 (-0.997, -0.351) | <0.001 | 0.243 (0.004, 0.481) | 0.046 | -0.432 (-0.837, -0.026) | 0.037 |
|  | WASH |  | 0.319 (-0.048, 0.111) | 0.433 | 0.003 (-0.046, 0.051) | 0.008 | 0.034 (-0.043, 0.111) | 0.382 |
|  | Dietary diversity |  | - | - | -0.021 (-0.084, 0.041) | 0.505 | -0.021 (-0.084, 0.041) | 0.505 |
|  | Household hunger |  | - | - | 0.000 (-0.039, 0.039) | 0.996 | 0.000 (-0.039, 0.039) | 0.996 |
| Ferritin (ug/L) | Dietary diversity |  | -0.020 (-0.080, 0.040) | 0.506 | - | - | -0.020 (-0.080, 0.040) | 0.506 |
|  | Household hunger |  | -0.012 (-0.052, 0.028) | 0.561 | - | - | -0.012 (-0.052, 0.028) | 0.561 |
|  | ln CRP (mg/L) |  | 0.094 (0.037, 0.152) | 0.001 | - | - | 0.094 (0.037, 0.152) | 0.001 |
|  | ln AGP (g/L) |  | 0.374 (0.113, 0.636) | 0.005 | - | - | 0.374 (0.113, 0.636) | 0.005 |
|  | WASH |  | -0.004 (-0.058, 0.051) | 0.895 | -0.016 (-0.045, 0.013) | 0.29 | -0.019 (-0.075, 0.036) | 0.492 |
| ln Retinol (umol/L) | Dietary diversity |  | 0.056 (0.003 (0.109) | 0.04 | - | - | 0.056 (0.003 (0.109) | 0.040 |
|  | Household hunger |  | -0.006 (-0.032, 0.019) | 0.626 | - | - | -0.006 (-0.032, 0.019) | 0.626 |
|  | ln CRP (mg/L) |  | -0.029 (-0.073, 0.015) | 0.191 | - | - | -0.029 (-0.073, 0.015) | 0.191 |
|  | ln AGP (g/L) |  | -0.108 (-0.310, 0.095) | 0.297 | - | - | -0.108 (-0.310, 0.095) | 0.297 |
|  | WASH |  | 0.000 (-0.050, 0.050) | 0.995 | 0.016 (-0.002, 0.035) | 0.078 | 0.017 (-0.032, 0.065) | 0.506 |
| ln Vitamin B12 (pmol/L) | Dietary diversity |  | 0.024 (-0.025, 0.072) | 0.337 | - | - | 0.024 (-0.025, 0.072) | 0.337 |
|  | Household hunger |  | -0.021 (-0.039, -0.004) | 0.017 | - | - | -0.021 (-0.039, -0.004) | 0.017 |
|  | ln CRP (mg/L) |  | -0.028 (-0.051, -0.006) | 0.015 | - | - | -0.028 (-0.051, -0.006) | 0.015 |
|  | ln AGP (g/L) |  | 0.227 (0.096, 0.358) | 0.001 | - | - | 0.227 (0.096, 0.358) | 0.001 |
|  | WASH |  | -0.002 (-0.051, 0.048) | 0.94 | 0.012 (-0.001, 0.024) | 0.06 | 0.010 (-0.035, 0.055) | 0.668 |
| ln Folate (nmol/L) | Dietary diversity |  | 0.020 (-0.008, 0.048) | 0.169 | - | - | 0.020 (-0.008, 0.048) | 0.169 |
|  | Household hunger |  | -0.018 (-0.040, 0.004) | 0.105 | - | - | -0.018 (-0.040, 0.004) | 0.105 |
|  | ln CRP (mg/L) |  | 0.001 (-0.026, 0.028) | 0.933 | - | - | 0.001 (-0.026, 0.028) | 0.933 |
|  | ln AGP (g/L) |  | 0.079 (-0.066, 0.224) | 0.288 | - | - | 0.079 (-0.066, 0.224) | 0.288 |
|  | WASH |  | -0.002 (-0.052, 0.048) | 0.942 | 0.011 (-0.001, 0.023) | 0.066 | 0.009 (-0.036, 0.055) | 0.690 |
| ln Zinc (umol/L) | Dietary diversity |  | 0.021 (-0.010, 0.051) | 0.179 | - | - | 0.021 (-0.010, 0.051) | 0.179 |
|  | Household hunger |  | -0.011 (-0.026, 0.003) | 0.128 | - | - | -0.011 (-0.026, 0.003) | 0.128 |
|  | ln CRP (mg/L) |  | 0.004 (-0.015, 0.022) | 0.717 | - | - | 0.004 (-0.015, 0.022) | 0.717 |
|  | ln AGP (g/L) |  | -0.161 (-0.284, -0.038) | 0.010 | - | - | -0.161 (-0.284, -0.038) | 0.010 |
|  | WASH |  | 0.005 (-0.023, 0.034) | 0.709 | 0.014 (0.001, 0.028) | 0.042 | 0.020 (-0.008, 0.048) | 0.166 |
| ln AGP (g/L) | WASH |  | -0.032 (-0.059, -0.005) | 0.019 | - | - | -0.032 (-0.059, -0.005) | 0.019 |
| ln CRP (mg/L) | WASH |  | -0.097 (-0.237, 0.042) | 0.172 | - | - | -0.097 (-0.237, 0.042) | 0.172 |
| Dietary diversity | WASH |  | 0.110 (0.033, 0.187) | 0.005 | - | - | 0.110 (0.033, 0.187) | 0.005 |
| Household hunger | WASH |  | -0.642 (-0.755, -0.530) | <0.001 | - | - | -0.642 (-0.755, -0.530) | <0.001 |
| 1 Values are β coefficients (95% CI). All adjusted for clustering. Adjusted analyses accounting for age of child in months. Estimates of zero are not shown. * p<0.05. ** p<0.01. *** p<0.001 | | | | | | | | |
| Fit statistics: CFI=0.938, TLI=0.742, RMSEA=0.064, SRMR=0.031 | | | | | | | | |

| Supplemental Table 11: Standardized direct, indirect, and total effects for path model in mothers of reproductive age living in Uttar Pradesh, using full information maximum likelihood (N=1238)^1^ | | | | | | | | | | |
| --- | --- | --- | --- | --- | --- | --- | --- | --- | --- | --- |
|  |  |  | **Standardized Coefficient** | | | | | | | |
| **Dependent variables** | **Predictors** |  | **Direct Effect** | **p-value** | **Indirect effect** | **p-value** | **Total effect** | **p-value** |  |  |
| Hemoglobin | Zinc |  | 0.054 | 0.133 | - | - | 0.054 | 0.133 |  |  |
|  | Folate |  | -0.098 | 0.059 | - | - | -0.098 | 0.059 |  |  |
|  | Vitamin B12 |  | 0.080 | 0.011 | - | - | 0.080 | 0.011 |  |  |
|  | Retinol |  | 0.044 | 0.154 | - | - | 0.044 | 0.154 |  |  |
|  | Ferritin |  | 0.527 | <0.001 | - | - | 0.527 | <0.001 |  |  |
|  | CRP |  | -0.052 | 0.173 | 0.067 | 0.005 | 0.014 | 0.756 |  |  |
|  | AGP |  | -0.010 | 0.848 | 0.046 | 0.273 | 0.036 | 0.605 |  |  |
|  | WASH |  | - | - | 0.022 | 0.387 | 0.022 | 0.387 |  |  |
|  | Dietary diversity |  | - | - | -0.001 | 0.988 | -0.001 | 0.988 |  |  |
|  | Household hunger |  | - | - | -0.023 | 0.444 | -0.023 | 0.444 |  |  |
|  |  |  |  |  |  |  |  |  |  |  |
| Ferritin | Dietary diversity |  | 0.012 | 0.834 | - | - | 0.012 | 0.834 |  |  |
|  | Household hunger |  | -0.050 | 0.354 | - | - | -0.050 | 0.354 |  |  |
|  | CRP |  | 0.134 | 0.004 | - | - | 0.134 | 0.004 |  |  |
|  | AGP |  | 0.079 | 0.248 | - | - | 0.079 | 0.248 |  |  |
|  | WASH |  | 0.008 | 0.865 | 0.038 | 0.156 | 0.046 | 0.232 |  |  |
| Retinol | Dietary diversity |  | 0.060 | 0.072 | - | - | 0.060 | 0.072 |  |  |
|  | Household hunger |  | -0.025 | 0.566 | - | - | -0.025 | 0.566 |  |  |
|  | WASH |  | 0.019 | 0.652 | 0.023 | 0.230 | 0.042 | 0.303 |  |  |
|  | CRP (mg/L) |  | 0.056 | 0.319 | - | - | 0.056 | 0.319 |  |  |
|  | AGP (g/L) |  | -0.084 | 0.176 | - | - | -0.084 | 0.176 |  |  |
| Vitamin B12 | Dietary diversity |  | 0.069 | 0.107 | - | - | 0.069 | 0.107 |  |  |
|  | Household hunger |  | 0.008 | 0.803 | - | - | 0.008 | 0.803 |  |  |
|  | CRP (mg/L) |  | 0.001 | 0.990 | - | - | 0.001 | 0.99 |  |  |
|  | AGP (g/L) |  | 0.103 | 0.042 | - | - | 0.103 | 0.042 |  |  |
|  | WASH |  | 0.059 | 0.136 | 0.013 | 0.379 | 0.059 | 0.136 |  |  |
| Folate | Dietary diversity |  | 0.121 | 0.008 | - | - | 0.121 | 0.008 |  |  |
|  | Household hunger |  | -0.057 | 0.156 | - | - | -0.057 | 0.156 |  |  |
|  | CRP (mg/L) |  | 0.091 | 0.003 | - | - | 0.091 | 0.003 |  |  |
|  | AGP (g/L) |  | -0.019 | 0.661 | - | - | -0.019 | 0.661 |  |  |
|  | WASH |  | 0.039 | 0.461 | 0.051 | 0.007 | 0.039 | 0.461 |  |  |
| Zinc | Dietary diversity |  | -0.058 | 0.276 | - | - | -0.058 | 0.276 |  |  |
|  | Household hunger |  | -0.033 | 0.529 | - | - | -0.033 | 0.529 |  |  |
|  | WASH |  | 0.011 | 0.686 | 0.006 | 0.831 | 0.011 | 0.686 |  |  |
|  | CRP |  | 0.049 | 0.29 | - | - | 0.049 | 0.29 |  |  |
|  | AGP |  | -0.039 | 0.470 | - | - | -0.039 | 0.470 |  |  |
| AGP | WASH |  | 0.037 | 0.315 | - | - | 0.037 | 0.315 |  |  |
| CRP | WASH |  | 0.111 | 0.039 | - | - | 0.111 | 0.039 |  |  |
| Dietary diversity | WASH |  | 0.178 | <0.001 | - | - | 0.178 | <0.001 |  |  |
| Household hunger | WASH |  | -0.360 | <0.001 | - | - | -0.360 | <0.001 |  |  |
| 1 Values are β coefficients (SE). All adjusted for clustering. Adjusted analyses accounting for age of child in months. Estimates of zero are not shown. * p<0.05. ** p<0.01. *** p<0.001 | | | | | | | | | | |
| Fit statistics: CFI=0.925, TLI=0.771, RMSEA=0.063, SRMR=0.034 | | | | | | | | |  |  |

| Supplemental Table 12: Unstandardized direct, indirect, and total effects for path model in mothers of reproductive age living in Uttar Pradesh, using full information maximum likelihood (N=1238)^1^ | | | | | | | |  |
| --- | --- | --- | --- | --- | --- | --- | --- | --- |
|  |  |  | **Unstandardized Coefficient (95% CI)** | | | | |  |
| **Dependent variables** | **Predictors** |  | **Direct Effect** | **p-value** | **Indirect effect** | **p-value** | **Total effect** | **p-value** |
| Hemoglobin (g/dL) | ln Zinc (umol/L) |  | 0.213 (-0.065, 0.492) | 0.133 | - | - | 0.213 (-0.065, 0.492) | 0.133 |
|  | ln Folate (nmol/L) |  | -0.257 (-0.523, 0.010) | 0.059 | - | - | -0.257 (-0.523, 0.010) | 0.059 |
|  | ln Vitamin B12 (pmol/L) |  | 0.228 (0.052, 0.403) | 0.011 | - | - | 0.228 (0.052, 0.403) | 0.011 |
|  | ln Retinol (umol/L) |  | 0.123 (-0.046, 0.291) | 0.154 | - | - | 0.123 (-0.046, 0.291) | 0.154 |
|  | Ferritin (ug/L) |  | 1.001 (0.809, 1.193) | <0.001 | - | - | 1.001 (0.809, 1.193) | <0.001 |
|  | ln CRP (mg/L) |  | -0.051 (-0.124, 0.022) | 0.173 | 0.065 (0.019, 0.110) | 0.005 | 0.014 (-0.074, 0.101) | 0.756 |
|  | ln AGP (g/L) |  | -0.044 (-0.497, 0.408) | 0.848 | 0.213 (-0.168, 0.595) | 0.273 | 0.169 (-0.472, 0.810) | 0.605 |
|  | WASH |  | - | - | 0.026 (-0.033, 0.084) | 0.387 | 0.026 (-0.033, 0.084) | 0.387 |
|  | Dietary diversity |  | - | - | -0.001 (-0.099, 0.097) | 0.988 | -0.001 (-0.099, 0.097) | 0.988 |
|  | Household hunger |  | - | - | -0.016 (-0.055, 0.024) | 0.444 | -0.016 (-0.055, 0.024) | 0.444 |
| Ferritin (ug/L) | Dietary diversity |  | 0.009 (-0.077, 0.096) | 0.834 | - | - | 0.009 (-0.077, 0.096) | 0.834 |
|  | Household hunger |  | -0.018 (-0.055, 0.020) | 0.354 | - | - | -0.018 (-0.055, 0.020) | 0.354 |
|  | ln CRP (mg/L) |  | 0.068 (0.022, 0.114) | 0.004 | - | - | 0.068 (0.022, 0.114) | 0.004 |
|  | ln AGP (g/L) |  | 0.193 (-0.135, 0.521) | 0.248 | - | - | 0.193 (-0.135, 0.521) | 0.248 |
|  | WASH |  | 0.005 (-0.054, 0.064) | 0.865 | 0.024 (-0.009, 0.057) | 0.156 | 0.029 (-0.019, 0.077) | 0.232 |
| ln Retinol (umol/L) | Dietary diversity |  | 0.032 (-0.003, 0.067) | 0.072 | - | - | 0.032 (-0.003, 0.067) | 0.072 |
|  | Household hunger |  | -0.006 (-0.027, 0.015) | 0.566 | - | - | -0.006 (-0.027, 0.015) | 0.566 |
|  | WASH |  | 0.008 (-0.027, 0.044) | 0.652 | 0.010 (-0.006, 0.026) | 0.230 | 0.018 (-0.016, 0.052) | 0.303 |
|  | CRP (mg/L) |  | 0.020 (-0.019, 0.058) | 0.319 | - | - | 0.020 (-0.019, 0.058) | 0.319 |
|  | AGP (g/L) |  | -0.141 (-0.344, 0.063) | 0.176 | - | - | -0.141 (-0.344, 0.063) | 0.176 |
| ln Vitamin B12 (pmol/L) | Dietary diversity |  | 0.036 (-0.008, 0.080) | 0.107 | - | - | 0.036 (-0.008, 0.080) | 0.107 |
|  | Household hunger |  | 0.002 (-0.012, 0.016) | 0.803 | - | - | 0.002 (-0.012, 0.016) | 0.803 |
|  | CRP (mg/L) |  | 0.000 (-0.036, 0.036) | 0.990 | - | - | 0.000 (-0.036, 0.036) | 0.990 |
|  | AGP (g/L) |  | 0.170 (0.006, 0.333) | 0.042 | - | - | 0.170 (0.006, 0.333) | 0.042 |
|  | WASH |  | 0.019 (-0.010, 0.049) | 0.194 | 0.006 (-0.007, 0.018) | 0.379 | 0.025 (-0.008, 0.058) | 0.136 |
| ln Folate (nmol/L) | Dietary diversity |  | 0.068 (0.018, 0.119) | 0.008 | - | - | 0.068 (0.018, 0.119) | 0.008 |
|  | Household hunger |  | -0.014 (-0.034, 0.006) | 0.156 | - | - | -0.014 (-0.034, 0.006) | 0.156 |
|  | CRP (mg/L) |  | 0.034 (0.012, 0.056) | 0.003 | - | - | 0.034 (0.012, 0.056) | 0.003 |
|  | AGP (g/L) |  | -0.034 (-0.184, 0.117) | 0.661 | - | - | -0.034 (-0.184, 0.117) | 0.661 |
|  | WASH |  | -0.006 (-0.052, 0.040) | 0.806 | 0.023 (0.007, 0.040) | 0.007 | 0.018 (-0.029, 0.064) | 0.461 |
| ln Zinc (umol/L) | Dietary diversity |  | -0.022 (-0.061, 0.017) | 0.276 | - | - | -0.022 (-0.061, 0.017) | 0.276 |
|  | Household hunger |  | -0.006 (-0.023, 0.012) | 0.529 | - | - | -0.006 (-0.023, 0.012) | 0.529 |
|  | ln CRP (mg/L) |  | 0.012 (-0.010, 0.034) | 0.290 | - | - | 0.012 (-0.010, 0.034) | 0.290 |
|  | ln AGP (g/L) |  | -0.046 (-0.172, 0.079) | 0.470 | - | - | -0.046 (-0.172, 0.079) | 0.470 |
|  | WASH |  | 0.002 (-0.020, 0.023) | 0.884 | 0.002 (-0.014, 0.017) | 0.831 | 0.003 (-0.013, 0.019) | 0.686 |
| ln AGP (g/L) | WASH |  | 0.009 (-0.009, 0.028) | 0.315 | - | - | 0.009 (-0.009, 0.028) | 0.315 |
| ln CRP (mg/L) | WASH |  | 0.137 (0.007, 0.267) | 0.039 | - | - | 0.137 (0.007, 0.267) | 0.039 |
| Dietary diversity | WASH |  | 0.143 (0.089, 0.196) | <0.001 | - | - | 0.143 (0.089, 0.196) | <0.001 |
| Household hunger | WASH |  | -0.644 (-0.753, -0.535) | <0.001 | - | - | -0.644 (-0.753, -0.535) | <0.001 |
| 1 Values are β coefficients (95% CI). All adjusted for clustering. Adjusted analyses accounting for age of child in months. Estimates of zero are not shown. * p<0.05. ** p<0.01. *** p<0.001 | | | | | | | |  |
| Fit statistics: CFI=0.925, TLI=0.771, RMSEA=0.063, SRMR=0.034 | | | | | | | | |

Supplemental Figure 1. Sampling flowchart for state survey in Uttar Pradesh^1^

^1^ CEB, Census enumeration block; PSC, preschool-age children; MRA, mothers of reproductive age

Supplemental Figure 2: Path model of the predictors of hemoglobin concentration in preschool-age children and mothers of reproductive age^1^

^1^AGP, alpha-1 acid glycoprotein; CRP, C-reactive protein; DD, dietary diversity score; RBC, red blood cell; WASH, water, sanitation, and hygiene.

Explanation of the theory development behind the model: We start with the nutritional causes of anemia, followed by environmental, inflammation, and genetic determinants. *Ferritin 🡪 Hemoglobin*: Ferritin is a measure of body stores. ^(1)^. Iron has often been cited as the primary cause of anemia. Iron is an integral element in the hemoglobin molecule, whereby Fe^2+^ binds to the protein protoporphyrin IX complex to form haem ^(2)^. When there is a depletion of iron stores, this compromises the supply of iron to body tissues, thereby reducing the production of hemoglobin^(3)^. *Zinc 🡪 Hemoglobin*: Zinc regulates erythropoiesis in the bone marrow. Zinc is a key component of GATA-1, an essential erythroid transcription factor ^(4)^. *Retinol 🡪 Hemoglobin*: Retinol plays a role in erythropoiesis. Its role in reducing anemia may come from its ability to increase iron absorption, to affect transferrin receptors and thus iron stores, stimulate erythroid precursors in bone marrow, and reduce susceptibility to infection.^(2, 5)^ *Vitamin B12 🡪 Hemoglobin*: vitamin B12 deficiency, like folate, may inhibit DNA synthesis and can result in megaloblastic macrocytic anemia ^(6)^. *RBC folate 🡪 Hemoglobin*: folic acid is needed for the formation of heme and is required for maturation of erythrocytes; it can also lead to changes in cell morphology and erythrocyte death ^(2)^. *Dietary diversity and food insecurity 🡪 nutritional biomarkers*: Dietary diversity reflects the types of foods consumed; the quality and quantity of micronutrients may depend on the types of foods eaten. Food insecurity may influence nutritional status by predicting the quantity and frequency of food consumption. *WASH 🡪 dietary diversity and food insecurity:* When there is poor sanitation and hygiene, there is a higher risk of microbial ingestion. Illnesses that result from microbial ingestion may reduce appetite and the diversity of foods consumed and reduce the absorption of nutrients from ingested food^(7)^. *Inflammation (malaria, CRP and AGP) 🡪 Hemoglobin*: Acute and chronic inflammation, as reflected by CRP and AGP, may be reflective of infection or disease, such as diarrhea, malaria, or intestinal parasites, which may have important effects on the concentration of hemoglobin ^(2, 8, 9)^. *Inflammation (malaria, CRP and AGP) 🡪 ferritin*: Ferritin levels are elevated during infection as a defense mechanism to deprive parasites and bacteria of iron for growth. Hence, accounting for changes in ferritin during inflammation prevents the underestimation of depleted iron stores^(10, 11)^. *WASH 🡪 inflammation, dietary diversity and food insecurity*: Many have documented the effects of clean water consumption, sanitation, and hygiene practices on infection and inflammation, with a particular influence on diarrheal disease, nutritional intake, as well as malnutrition ^(7)^. *β-thalassemia 🡪 ferritin and hemoglobin*: β-thalassemia is a genetic hemoglobin disorder which can results in decreased hemoglobin production. In patients with β-thalassemia, ferritin is influenced by ineffective erythropoiesis, despite sometimes iron overload. The amount of downregulation may depend on the severity (minor, intermedia, major) of β-thalassemia^(12)^.

**References**

1. Pasricha S-R, Atkinson SH, Armitage AE *et al.* (2014) Expression of the iron hormone hepcidin distinguishes different types of anemia in African children. *Science translational medicine* 6, 235re233-235re233.

2. Balarajan Y, Ramakrishnan U, Ozaltin E *et al.* (2011) Anaemia in low-income and middle-income countries. *Lancet (London, England)* 378, 2123-2135.

3. Clark SF (2008) Iron deficiency anemia. *Nutrition in clinical practice* 23, 128-141.

4. Ferreira R, Ohneda K, Yamamoto M *et al.* (2005) GATA1 function, a paradigm for transcription factors in hematopoiesis. *Molecular and cellular biology* 25, 1215-1227.

5. Fishman SM, Christian P West KP (2000) The role of vitamins in the prevention and control of anaemia. *Public health nutrition* 3, 125-150.

6. Langan RC & Goodbred AJ (2017) Vitamin B12 Deficiency: Recognition and Management. *American family physician* 96, 384-389.

7. Ngure FM, Reid BM, Humphrey JH *et al.* (2014) Water, sanitation, and hygiene (WASH), environmental enteropathy, nutrition, and early child development: making the links. *Annals of the New York Academy of Sciences* 1308, 118-128.

8. Engle-Stone R, Aaron GJ, Huang J *et al.* (2017) Predictors of anemia in preschool children: Biomarkers Reflecting Inflammation and Nutritional Determinants of Anemia (BRINDA) project. *The American journal of clinical nutrition* 106, 402s-415s.

9. Wirth JP, Woodruff BA, Engle-Stone R *et al.* (2017) Predictors of anemia in women of reproductive age: Biomarkers Reflecting Inflammation and Nutritional Determinants of Anemia (BRINDA) project. *The American journal of clinical nutrition* 106, 416s-427s.

10. Kernan KF & Carcillo JA (2017) Hyperferritinemia and inflammation. *International immunology* 29, 401-409.

11. Namaste SM, Aaron GJ, Varadhan R *et al.* (2017) Methodologic approach for the Biomarkers Reflecting Inflammation and Nutritional Determinants of Anemia (BRINDA) project. *The American journal of clinical nutrition* 106, 333s-347s.

12. Ganz T (2013) Systemic iron homeostasis. *Physiological reviews* 93, 1721-1741.
